# Off-Target Expression of Cre-Dependent Adeno-Associated Viruses in Wild-Type C57BL/6J Mice

**DOI:** 10.1523/ENEURO.0363-21.2021

**Published:** 2021-11-24

**Authors:** Justin J. Botterill, Abdessattar Khlaifia, Brandon J. Walters, Mark A. Brimble, Helen E. Scharfman, Maithe Arruda-Carvalho

**Affiliations:** 1 Department of Psychology, University of Toronto Scarborough, Toronto, Ontario M1C 1A4, Canada; 2 Department of Cell & Systems Biology, University of Toronto Mississauga, Mississauga, Ontario L5L 1C6, Canada; 3 Department of Surgery, St. Jude Children’s Research Hospital, Memphis, Tennnessee 38105; 4 Center for Dementia Research, The Nathan Kline Institute for Psychiatric Research, Orangeburg, New York 10962; 5 Departments of Child & Adolescent Psychiatry, Neuroscience & Physiology, and Psychiatry, and New York University Neuroscience Institute, New York University Langone Health, New York, New York 10016; 6 Department of Cell and Systems Biology, University of Toronto, Toronto, Ontario M5S 3G5, Canada

**Keywords:** Immunofluorescence, antibody amplification, double inverted open reading frame, fear conditioning, c-Fos, Cre/loxP, DREADDs

## Abstract

Adeno-associated viruses (AAVs) are a commonly used tool in neuroscience to efficiently label, trace, and/or manipulate neuronal populations. Highly specific targeting can be achieved through recombinase-dependent AAVs in combination with transgenic rodent lines that express Cre-recombinase in specific cell types. Visualization of viral expression is typically achieved through fluorescent reporter proteins (e.g., GFP or mCherry) packaged within the AAV genome. Although nonamplified fluorescence is usually sufficient to observe viral expression, immunohistochemical amplification of the fluorescent reporter is routinely used to improve viral visualization. In the present study, Cre-dependent AAVs were injected into the neocortex of wild-type C57BL/6J mice. While we observed weak but consistent nonamplified off-target double inverted open reading frame (DIO) expression in C57BL/6J mice, antibody amplification of the GFP or mCherry reporter revealed notable Cre-independent viral expression. Off-target expression of DIO constructs in wild-type C57BL/6J mice occurred independent of vendor, AAV serotype, or promoter. We also evaluated whether Cre-independent expression had functional effects via designer receptors exclusively activated by designer drugs (DREADDs). The DREADD agonist C21 (compound 21) had no effect on contextual fear conditioning or c-Fos expression in DIO-hM3Dq-mCherry^+^ cells of C57BL/6J mice. Together, our results indicate that DIO constructs have off-target expression in wild-type subjects. Our findings are particularly important for the design of experiments featuring sensitive systems and/or quantitative measurements that could be negatively impacted by off-target expression.

## Significance Statement

Adeno-associated viruses (AAVs) are widely used in neuroscience because of their safety and ease of use. Combined with specific promoters, Cre/loxP, and stereotaxic injections, highly specific targeting of cells and circuits within the brain can be achieved. In the present study, we injected Cre-dependent AAVs into wild-type C57BL/6J mice and found Cre-independent viral expression of AAVs encoding mCherry, GFP, or hM3Dq following immunohistochemical amplification of the fluorescent reporter protein. Importantly, we observed no functional effects of the Cre-independent expression in the hippocampus, as C21 (compound 21) had no detectable effect on double inverted open reading frame (DIO)-hM3Dq-mCherry-infected neurons in C57BL/6J mice. Given the widespread use of DIO recombinant AAVs by the neuroscience community, our data support careful consideration when using DIO constructs in control animals.

## Introduction

A main goal of neuroscience is to understand the roles of specific cell types and circuits underlying neurodevelopment, behavior, and disease. Adeno-associated virus (AAV) represents a powerful tool for neuroscientists to address these questions via labeling and manipulating cell types and circuits. AAV is a dependoparvovirus comprising a small 4.7 kb single-stranded DNA genome with an unenveloped icosahedral capsid (Grieger and Samulski, 2005; Betley and Sternson, 2011; Haery et al., 2019; Haggerty et al., 2020). Recombinant AAVs (rAAVs) used in research and clinical applications are modified from wild-type (WT) AAVs and use an expression cassette to drive transgene expression. The rAAV expression cassette typically consists of a promoter, transgene, and polyadenylation signal flanked by inverted terminal repeats (ITRs; Saunders and Sabatini, 2015). A major advantage of rAAVs is their durable transgene expression (months to years) and limited pathogenic profile (Naso et al., 2017; Haery et al., 2019; Haggerty et al., 2020).

The Cre/loxP system is a powerful site-specific recombinase used to insert, delete, or invert DNA sequences between loxP sites (Sauer and Henderson, 1988; Sengupta et al., 2017; Fischer et al., 2019). Using the Cre/loxP system, discrete cell populations can be targeted through a combination of transgenic mice and viral injections. Using this method, rodents are genetically modified to express Cre in specific cell types, and therefore the injection of Cre-dependent constructs should only recombine in Cre-expressing cells within the injected area. Double inverted open reading frame (DIO) constructs are a common method to achieve Cre-dependent activation of genes. DIO constructs rely on two pairs of recombination-incompatible lox sites (loxP and lox2722) that surround the transgene, which is in the inverse orientation. However, in the presence of Cre, the DIO cassette is reverted, allowing the expression of the transgene (Fenno et al., 2011). DIO cassettes are widely used because DIO is considered to have low off-target expression (Fischer et al., 2019) because of the transgene being in the incorrect orientation. Additionally, DIO is much smaller than other constructs with a similar goal, facilitating its use in AAVs.

Visualization of rAAV expression is typically achieved with fluorescent reporter proteins; either fused to a transgene of interest or inserted into its own reading frame (Smith et al., 2016). Fluorescent reporters exhibit relatively strong and permanent expression in transduced neurons and depending on the method used can reveal expression in dendrites or axons (Betley and Sternson, 2011; Saleeba et al., 2019). The fluorescent reporter can also be inserted between loxP sites to allow for Cre-dependent expression of fluorescence signal (Betley and Sternson, 2011; Saunders and Sabatini, 2015; Saleeba et al., 2019). However, a limitation of fluorescent reporters is that expression can be weak in certain applications. For example, fluorescence can decline substantially following exposure to fixatives or high temperatures during tissue processing (Alkaabi et al., 2005). To circumvent weak rAAV fluorescence *ex vivo*, many studies amplify expression with antibodies against reporter proteins (e.g., GFP, mCherry) to improve visualization of fluorescence expression (Deverman et al., 2016; McGlinchey and Aston-Jones, 2018; Murata and Colonnese, 2020; Iwasaki and Ikegaya, 2021). Subjects that lack Cre are often used as controls for the behavioral or cellular effects of Cre-dependent viruses (Alexander et al., 2018; Bonaventura et al., 2019; Mahler et al., 2019), under the premise that these constructs limit expression to Cre^+^ cells.

In the present study, we found consistent Cre-independent expression of DIO constructs in C57BL/6J mice injected across different brain regions. While Cre-dependent rAAVs showed minimal nonamplified fluorescence in brain sections of WT C57BL/6J mice, fluorescence signal amplification revealed numerous positive cells within the region of viral infection. To address whether the amplified fluorescence signal had functional effects, we used the Cre-dependent designer receptors exclusively activated by designer drugs (DREADDs) construct hM3Dq-mCherry, which is a modified human muscarinic M3 receptor that promotes neuronal excitation when activated (Roth, 2016). We found no detectable effect of the hM3Dq agonist compound 21 (C21) on fear behavior or immediate early gene activity in the hippocampus of WT C57BL/6J mice. Our results have important implications for the use of DIO constructs in control subjects, particularly in sensitive circuits or studies focusing on quantitative analyses such as cell counting or evaluating fluorescence signal.

## Materials and Methods

### Animals

Adult male and female mice, 2–6 months of age, were used for all experiments. For experiments testing Cre-dependent viral expression in mice lacking Cre-recombinase, we used WT C57BL/6J mice (The Jackson Laboratory). Tyrosine hydroxylase-Cre (*TH-Cre*; a gift from Jonathan Britt, McGill University, Montreal, QC, Canada; Lindeberg et al., 2004) and parvalbumin-Cre (*PV-Cre*; The Jackson Laboratory) mice were used in a subset of experiments and genotyping for these lines was done in-house using standard PCR protocols. Mice were bred in-house and maintained on a 12 h light/dark cycle (lights on at 7:00 A.M.) with access to food and water *ad libitum*. Mice were housed in standard laboratory cages that contained corn cob bedding and a polycarbonate igloo shelter (Bio-Serv). Offspring were weaned with same-sex siblings on postnatal day 21 (two to five mice per cage). All experiments were performed during the light phase of the light/dark cycle. All animal procedures were approved by the Animal Care Committee at the University of Toronto. Experimenters were blinded for all quantitative analyses.

### Stereotaxic surgery and viral injections

Mice underwent stereotaxic surgery between 2-5 months of age. Briefly, mice were injected intraperitoneally with a combination of ketamine (100 mg/kg) and xylazine (5 mg/kg) to induce anesthesia. Once anesthetized, the head was shaved and swabbed with iodine followed by 70% ethanol. Tear gel (Alcon) was applied to the eyes to prevent dehydration. Mice were then secured in a rodent stereotaxic apparatus (Stoelting) using ear bars. Body temperature was maintained throughout surgery with a heating blanket. An incision was made down the midline of the scalp using a scalpel, the connective tissue was excised, and then the skull was cleaned with sterile PBS, pH 7.4. An autoclaved cotton-tip applicator was briefly submerged in 30% hydrogen peroxide and gently applied to the skull surface to identify bregma. Using bregma as a reference point, craniotomies were made over the left medial prefrontal cortex (mPFC; +1.9 mm anteroposterior, 0.3 mm mediolateral), left anterior hippocampus (−2.1 mm anteroposterior and −1.25 mm mediolateral), left posterior hippocampus (−3.05 mm anteroposterior, −2.35 mm mediolateral), or ventral tegmental area (VTA; −3.15 mm anteroposterior, ±0.45 mm mediolateral). Experiments targeting the mPFC used a single viral injection, whereas dual viral injections were administered for the hippocampus (anterior and posterior) and VTA (bilateral) experiments.

Virus was delivered using a 500 nl Neuros Syringe (catalog #65457–02, Hamilton) attached to the stereotaxic apparatus with a probe holder (catalog #751873, Harvard Apparatus). The syringe was positioned above each craniotomy, and the needle was lowered into the mPFC (−2.3 mm below skull surface), hippocampus (−1.95 mm anterior, −2.5 mm posterior below skull surface), or ventral tegmental area (−4.5 mm below skull surface). For each injection, 0.2 μl of virus was injected at a rate of 0.06 μl/min. The following viral constructs were used: AAV5-EF1a-DIO-enhanced yellow fluorescent protein [eYFP; ≥4 × 10^12^ vector genomes (vg)/ml; UNC Core, Chapel Hill, NC], AAV5-EF1a-DIO-mCherry (≥7 × 10^12^ vg/ml; UNC Core), AAV8-hSyn-DIO-hM3D(Gq)-mCherry (≥5 × 10^12^ vg/ml; UNC Core), or AAV5-hSyn-DIO-hM4D(Gi)-mCherry (≥8 × 10^12^ vg/ml; catalog #44362, Addgene). The needle remained in place for an additional 5 min after each injection to allow for diffusion of the virus, and then the needle was slowly removed from the brain. Ketoprofen (1 mg/kg, s.c.) was injected ∼30 min before the end of surgery to reduce discomfort. The skull was cleaned with sterile PBS, and the scalp was sutured with Vetbond tissue adhesive (3M). Mice were injected with 0.7 ml of warmed physiological saline at the end of surgery to support hydration. Mice were then transferred into a clean cage located on a heating blanket. Mice were returned to their colony room once fully ambulatory. Ketoprofen (1 mg/kg, s.c.) was administered 24 and 48 h after surgery to reduce postsurgical discomfort.

### Contextual fear conditioning

Contextual fear conditioning was selected as a behavioral assay because of previous reports indicating that context fear is sensitive to manipulations of hippocampal activity (Krueger et al., 2020; Botterill et al., 2021). Mice underwent a postsurgical recovery period of 2 weeks before behavioral testing. Mice were transferred to a dedicated procedures room and injected with C21 (1.5 mg/kg i.p., 0.2 mg/ml dissolved in 0.9% NaCl; Hello Bio) 1 h before fear training. Mice underwent contextual fear conditioning as previously described (Arruda-Carvalho et al., 2011; Guskjolen et al., 2018). Briefly, mice were individually placed in a stainless steel fear-conditioning apparatus (32 cm wide, 25.5 cm high, 25.5 cm deep) that contained shock grid floors (36 rods, 2 mm diameter). The fear-conditioning apparatus was located inside a sound-attenuated chamber (63.5 cm wide, 36.8 cm high, 74.9 cm deep; catalog #NIR-022MD, Med Associates). A 2 min acclimation period was used to assess baseline behavior. Footshocks (0.5 mA, 2 s duration) were delivered 120, 180, 240, 300, and 360 s after mice were placed in the chamber. Mice remained in the chamber for 60 s after the final footshock and were then returned to their home cage. Mice were returned to the colony housing room and left undisturbed until the context test on the following day. Contextual fear memory was assessed 24 h after the training session. Mice were returned to the same fear-conditioning chamber as the previous day in the absence of footshocks and freezing behavior was evaluated over 8 min. Notably, C21 was not administered before testing.

Conditioned freezing was identified by the absence of movement except those necessary for respiration (Blanchard and Blanchard, 1972; Fanselow, 1980). Freezing behavior was scored automatically using the Med Associates VideoFreeze software.

### Perfusions and sectioning

Mice were euthanized 2–3 weeks after surgery to evaluate viral expression. Subjects were injected with Avertin (250 mg/kg, i.p.), and, once under deep anesthesia, transcardially perfused with 15 ml of room temperature saline, followed by 15 ml of cold 4% paraformaldehyde (PFA). The brains were extracted and stored overnight at 4°C in 4% PFA. The brains were sectioned at 50 μm in the coronal plane with a vibratome (model VT1000, Leica) and stored at −20°C in a cryoprotectant solution comprised of 60% glycerol and 0.01% sodium azide in 0.1 m PBS.

### Immunofluorescence

Immunofluorescence staining was performed on free-floating sections. Sections were washed in 0.1 m PBS (3 × 5 min each) and then incubated in blocking solution composed of 5% normal goat serum and 0.25% Triton X-100 in 0.1 m PBS for 30 min. Amplification of the viral signal was achieved by incubating sections with polyclonal rabbit anti-mCherry (1:2000; catalog #ab167453, Abcam; RRID:AB_2571870) or polyclonal chicken anti-GFP (1:2000; catalog #ab13970, Abcam; RRID:AB_300798) primary antibodies diluted in blocking solution. Sections were incubated with the primary antibodies overnight at 4°C on a rotary shaker under gentle agitation. On the following morning, sections were incubated in goat anti-rabbit Alexa Fluor 568 (1:500; catalog #A11011, Thermo Fisher Scientific; RRID:AB_143157) or goat anti-chicken Alexa Fluor 488 (1:500; catalog #A11039, Thermo Fisher Scientific; RRID:AB_2534096) secondary antibodies for 2 h. Sections were then counterstained with Hoechst 33342 stain (1:2000 diluted in 0.1 m PBS; Thermo Fisher Scientific). Sections were then rinsed in 0.1 m PBS, mounted onto gelatin-coated slides, air dried for 30 min, and coverslipped with Citifluor anti-fade mounting medium (catalog #17970, Electron Microscopy Sciences).

A subset of tissue was processed for mCherry and c-Fos using tyramide signal amplification (TSA). Briefly, sections were rinsed in 0.1 m PBS, followed by 1% H_2_O_2_ in 0.1 m PBS to quench endogenous peroxidase activity. Sections were then incubated overnight at 4°C with polyclonal rabbit anti-c-Fos (catalog #226003, Synaptic Systems; RRID:AB_2231974) and monoclonal rat anti-mCherry (catalog #M11217, Thermo Fisher Scientific; RRID:AB_2536611) primary antibodies in 0.1 m Tris-buffered saline containing 0.5% Roche Blocking Reagent (catalog #11096176001; Sigma-Aldrich). On the following day, sections were incubated in goat anti-rat Alexa Fluor 568 secondary antibody (1:500; catalog #A11077, Thermo Fisher Scientific; RRID:AB_2534121) and donkey anti-rabbit horseradish peroxidase-conjugated secondary antibody (1:500; catalog #711–036-152, Jackson ImmunoResearch; RRID:AB_2340590) for 1 h each. Next, TSA was performed using fluorescein tyramide (1:100) diluted in 0.1 m borate buffer containing 0.01% H_2_O_2_ solution. Sections were counterstained with Hoechst 33342 stain (1:2000), mounted onto slides, air dried, and coverslipped with anti-fade mounting medium as described above.

### Diaminobenzidine staining

Immunohistochemistry for bright-field microscopy was performed using standard protocols (Koshimizu et al., 2021). Sections were rinsed in 0.1 m PBS, endogenous peroxidase activity was quenched with 0.3% H_2_O_2_, and blocked in 5% normal goat serum. Sections were incubated with polyclonal rabbit anti-mCherry primary antibody (1:8000) diluted in blocking solution overnight at 4°C. On the following day, sections were incubated in biotinylated goat anti-rabbit secondary antibody (1:500; catalog #BA-1000, Vector Laboratories; RRID:AB_2313606) and avidin–biotin complex (1:500; catalog #PK-6100, Vector Laboratories; RRID:AB_2336819). Immunoreactivity was visualized by incubating sections in 0.5 mg/ml diaminobenzidine (DAB; Sigma-Aldrich), 40 μg/ml ammonium chloride (Sigma-Aldrich), 25 mg/ml (D+)-glucose (Sigma-Aldrich), and 3 μg/ml glucose oxidase (Sigma-Aldrich) for ∼5 min. Sections were mounted onto gelatin-coated slides and allowed to dry overnight. Sections were then dehydrated using a graded alcohol series (70, 95, 100%), cleared with xylenes, and coverslipped with Permount mounting medium (Electron Microscopy Sciences).

### Image acquisition

Images were acquired with a Nikon Eclipse Ni-U epifluorescence microscope running NIS-elements software (version 5.11.03; Nikon). Immunofluorescence was visualized with an LED illumination system (X-Cite 120 LED Boost, Excelitas Technologies) and captured with a Nikon DS-Qi2 digital camera. Immunofluorescence images were acquired using Plan Fluor 4×, Plan-Apochromat 10× differential interference contrast (DIC) N1 or Plan Fluor 20× DIC N2 objectives. Bright-field images were acquired with a 10× objective on an Olympus BX61 microscope. Figures were made using Photoshop version 22.5 (Adobe). When brightness and/or contrast adjustments were made in a figure, these changes were made equally to all photomicrographs.

### Quantification

Cell counts were performed manually using ImageJ software (version 1.53e) by experimenters blinded to treatment conditions. For cell counts in the hippocampus, counts were performed on a minimum of five sections per subject that spanned the rostral–caudal extent of the hippocampus. For cell counts in the mPFC, approximately three to four sections were counted per subject because of the smaller number of available sections for this region. Cell counts were performed for both the injected and noninjected hemispheres for each subject. The average number of cells per section was calculated by summing the total number of cells counted in the injected or noninjected hemisphere and dividing by the number of sections that were analyzed.

Quantification of c-Fos^+^ and mCherry^+^ was performed as previously described (Botterill et al., 2021). Briefly, we evaluated the percent colocalization of c-Fos and mCherry by counting the number of c-Fos^+^mCherry^+^ cells divided by the total number of mCherry^+^ cells for each mouse. Cell counts were performed on an average of five sections per mouse. Double-labeled cells were defined as cells with a yellow center (c-Fos and mCherry) surrounded by a red cytoplasm (mCherry).

### Statistical analysis

All results are presented as the mean ± SEM. Statistical comparisons were made using Prism 9.0 (GraphPad Software) with statistical significance (*p* < 0.05) denoted on all graphs with an asterisk. Comparisons of independent groups were made using two-tailed unpaired *t* tests or one-way ANOVA. Two-way repeated-measures ANOVAs were used to analyze parametric data with multiple comparisons followed by Tukey’s *post hoc* test with corrections for multiple comparisons when appropriate. Normality of parametric datasets were confirmed by the D’Agostino and Pearson normality test (Prism 9.0). Nonparametric datasets were analyzed with a Mann–Whitney *U* test. Potential sex differences were examined for each dataset and indicated no significant differences between male and female mice (all *p* values > 0.154). Male and female mice were therefore pooled for each dataset, but for transparency, all graphs show individual data points for male (dotted) and female (clear) mice.

## Results

### Fluorescence signal amplification of DIO constructs in Cre^+^ and WT C57BL/6J mice

**Figure 1.**
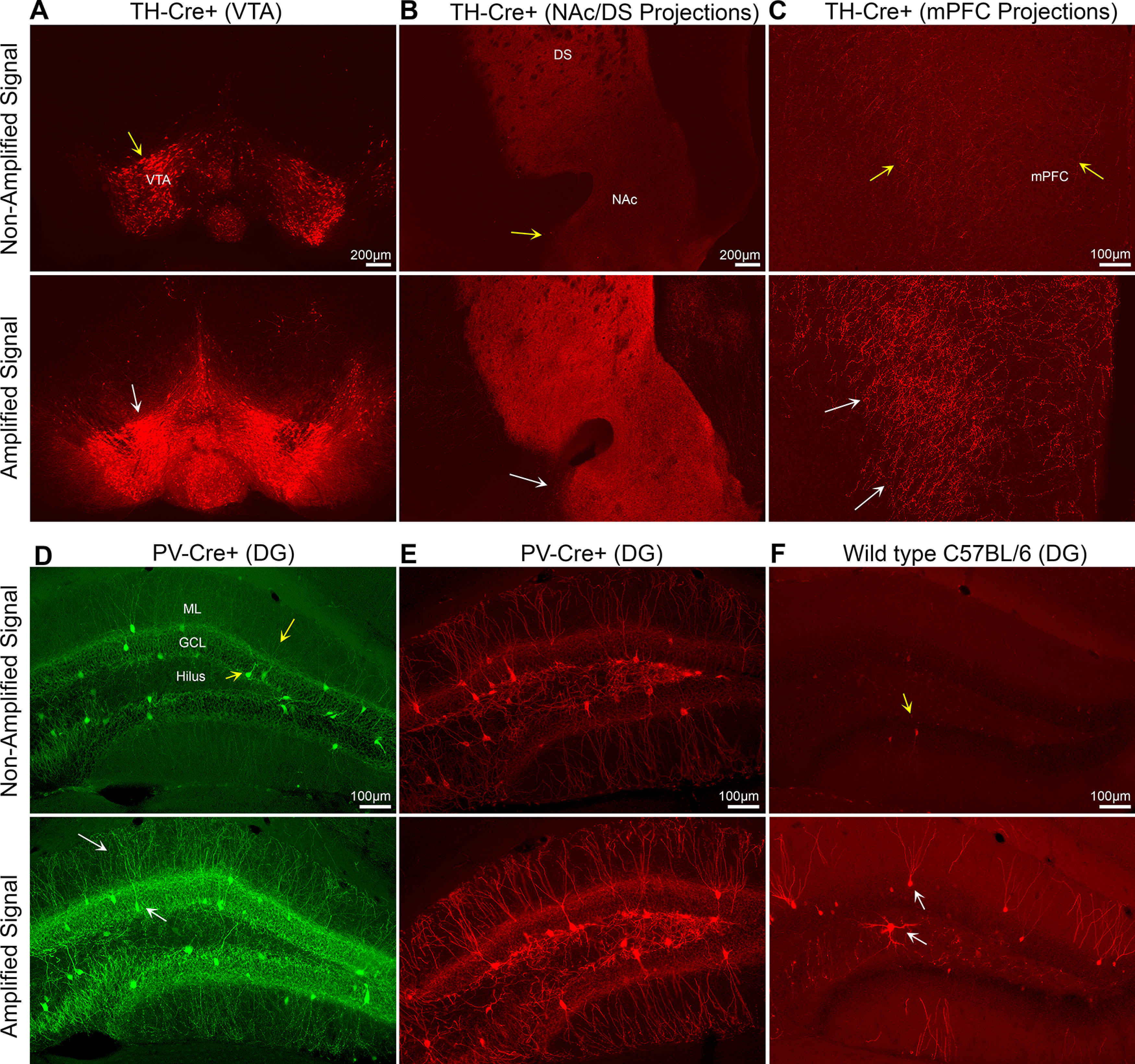
Antibody amplification of Cre-dependent viral expression. ***A***, Representative images from a *TH-Cre* mouse injected in the VTA with AAV5-EF1a-DIO-mCherry show a similar pattern of expression between nonamplified and amplified fluorescence (yellow and white arrows). ***B***, Long-range VTA–NAc/DS projections are easier to visualize following mCherry amplification (yellow vs white arrow). ***C***, Similarly, nonamplified fluorescence of VTA to mPFC projections was generally weak (yellow arrows), and the fluorescence signal was significantly improved following mCherry amplification (white arrows). ***D***, ***E***, Representative images from *PV-Cre* mice injected with AAV5-EF1a-DIO-EYFP (***D***) or AAV5-EF1a-DIO-mCherry (***E***). The nonamplified fluorescence signal was similar between eYFP and mCherry constructs. Moreover, fluorescence signal amplification is similar to the nonamplified signal (yellow arrows) but is brighter and easier to visualize (white arrows), especially the dendrites in the ML. ***F***, Representative images from a C57BL/6J mouse injected with AAV5-EF1a-DIO-mCherry show minimal nonamplified fluorescence (yellow arrow). Remarkably, amplification of adjacent sections from the same mouse revealed mCherry expression within the DG (white arrows). Scale bars: Panels ***A***–***B***: 200 μm; Panels ***C***–***F***: 100 μm.

First, we evaluated nonamplified and amplified fluorescence of a DIO-mCherry construct in the *TH-Cre* mouse line, which labels dopaminergic neurons in midbrain structures such as the VTA (Lammel et al., 2015; Popescu et al., 2016). AAV5-EF1a-DIO-mCherry was injected bilaterally into the VTA of *TH-Cre* mice (Fig. 1*A*). Near the injection site, nonamplified and amplified fluorescence showed a pattern of fluorescence consistent with previous reports (Lammel et al., 2015), but mCherry amplification produced a substantial increase in fluorescence signal. We also evaluated long-range projections from the VTA to nucleus accumbens (NAc), dorsal striatum (DS), and mPFC. The NAc and DS showed a moderate amount of nonamplified fluorescence, whereas mCherry^+^ terminals in the mPFC were only slightly greater than background fluorescence. In contrast, amplifying the fluorescent signal revealed bright fluorescence in the NAc-DS and numerous mCherry^+^ terminals in the mPFC (Fig. 1*B*,*C*), in a pattern consistent with previous reports (Stuber et al., 2010; Lammel et al., 2015; Popescu et al., 2016; Ellwood et al., 2017).

We further compared nonamplified and amplified fluorescence in the hippocampus, a brain region widely studied and often targeted with DIO constructs. Using *PV-Cre* mice, which express Cre in parvalbumin interneurons, we targeted the dentate gyrus (DG) subfield of the hippocampus because of its well documented PV expression (Freund and Buzsáki, 1998; Pelkey et al., 2017). *PV-Cre* mice were injected with AAV5-EF1a-DIO-eYFP or AAV5-EF1a-DIO-mCherry (Fig. 1*D*,*E*). In both cases, the nonamplified signal in the DG was characterized by bright fluorescence in somata and weaker fluorescence in fine processes, consistent with the overall patterns of parvalbumin immunoreactivity reported previously (Zou et al., 2016; Foggetti et al., 2019). Antibody amplification of GFP or mCherry resulted in a brighter immunofluorescence signal, especially in fine processes, such as dendrites extending into the molecular layer (ML; Fig. 1*D*,*E*). The results of the *TH-Cre*^+^ and *PV-Cre*^+^ experiments suggest that fluorescence signal amplification produces immunofluorescence expression that is faithful to nonamplified viral expression, but advantageous for visualizing cells or terminals with weak fluorescence.

Control experiments were also performed where AAV5-EF1a-DIO-mCherry was injected into the DG of WT C57BL/6J mice. Compared with the substantial fluorescence signal observed in the DG of Cre^+^ mice, we observed minimal nonamplified fluorescence in control mice (Fig. 1*F*). This observation is consistent with the requirement of Cre-recombinase for transgene expression and low “leak” with DIO constructs (Schnütgen et al., 2003; Atasoy et al., 2008; Saunders and Sabatini, 2015). However, amplification of DIO-mCherry revealed immunofluorescence within the DG of control C57BL/6J mice (Fig. 1*F*). The majority of amplified mCherry^+^ cells appeared to be granule cells (GCs), which reside in the principal cell layer of the DG known as the granule cell layer (GCL) and extend dendrites into the ML. We also observed sparse labeling of mCherry^+^ boutons in the hilus, consistent with expression of mCherry in dentate GC mossy fibers. Sparse labeling of large hilar cells was also observed. These data show that fluorescence signal amplification revealed notable off-target expression in mice lacking Cre-recombinase.

### Nonamplified expression of DIO constructs in WT C57BL/6J mice

**Figure 2.**
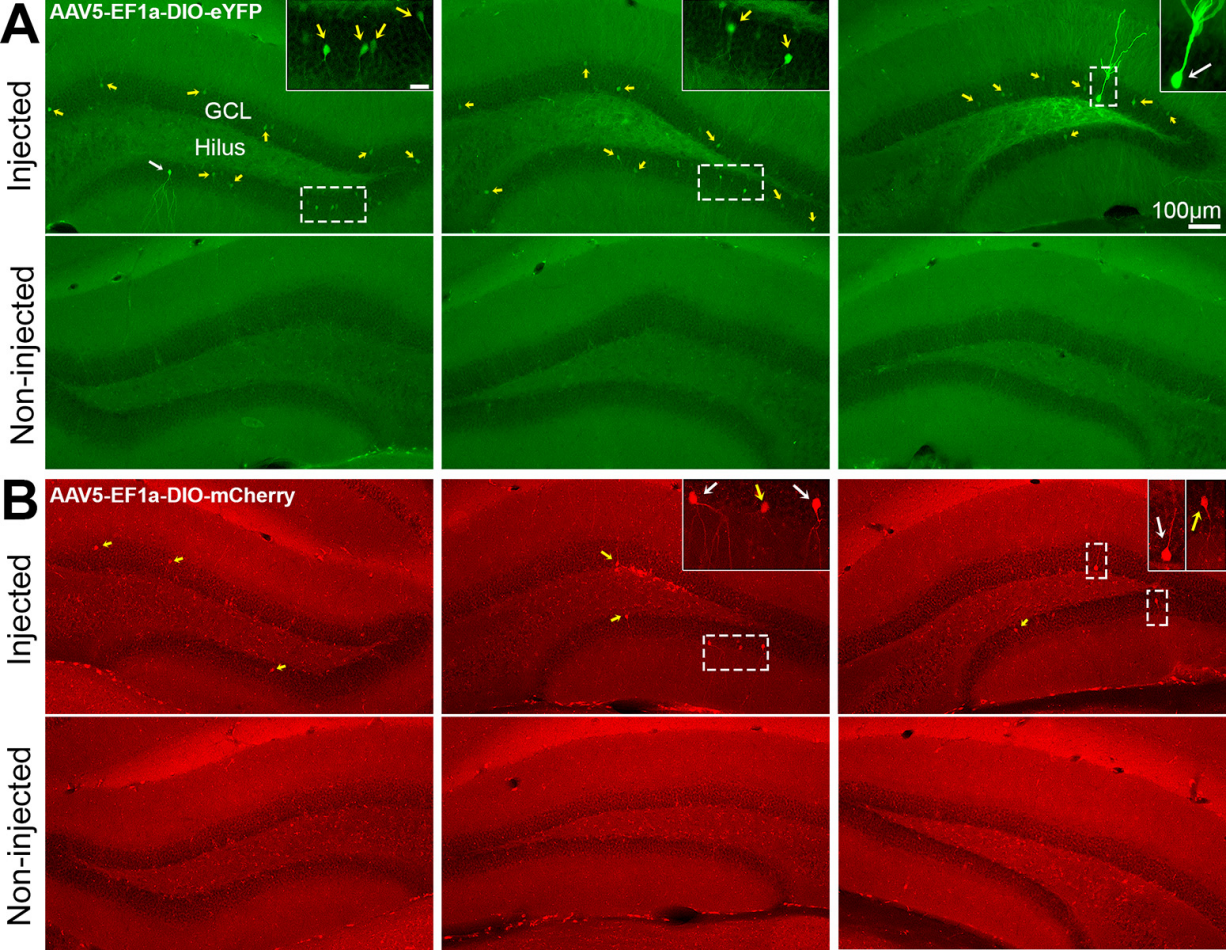
Nonamplified fluorescence of DIO constructs in WT C57BL/6J mice. ***A***, ***B***, Representative photomicrographs of nonamplified fluorescence signal in C57BL/6J mice injected with AAV5-EF1a-DIO-eYFP (***A***) or AAV5-EF1a-DIO-mCherry (***B***). Nonamplified immunofluorescence was generally weak and primarily restricted to the soma (yellow arrows; see insets) of the injected hemisphere only. We hypothesize that the weak nonamplified immunofluorescence in these cells is significantly enhanced after antibody amplification. In addition, a very small number of cells with bright immunofluorescence throughout the cell body and its processes were observed (white arrows; see insets). Scale bars: 10× objective, 100 μm; 20× objective, insets, 25 μm.

Next, we evaluated nonamplified fluorescence of DIO constructs in C57BL/6J mice to gain a better understanding of the off-target expression observed following fluorescence amplification. Non-amplified sections of C57BL/6J mice injected with AAV5-EF1a-DIO-eYFP or AAV5-EF1a-DIO-mCherry showed very few bright GFP^+^ or mCherry^+^ cells, respectively (Fig. 2). This finding is consistent with the notion that Cre is required to drive transgene expression, but Cre-independent expression is possible (Fischer et al., 2019; Morceau et al., 2019). Specifically, commercial vendors warn that recombination of loxP sites may occur during DNA amplification and viral production and result in Cre-independent transgene expression. However, this is thought to occur in a small number of viral particles (e.g., <1%) and therefore represent a minor source of off-target expression. Indeed, the few cells with bright fluorescence cannot explain the numerous cells we observed following fluorescence amplification. We found that increasing the exposure time and using higher-power objectives (e.g., 20×) revealed numerous cells with weak fluorescence primarily restricted to the soma (Fig. 2, insets). Importantly, cells with weak fluorescence were only observed in the injected hemisphere. We hypothesize that these numerous but weakly labeled cells express low levels of the viral transgene (e.g., GFP or mCherry) and become strongly labeled following fluorescence signal amplification.

### Fluorescence signal amplification of AAV5-EF1a-DIO-mCherry in WT C57BL/6J mice

**Figure 3.**
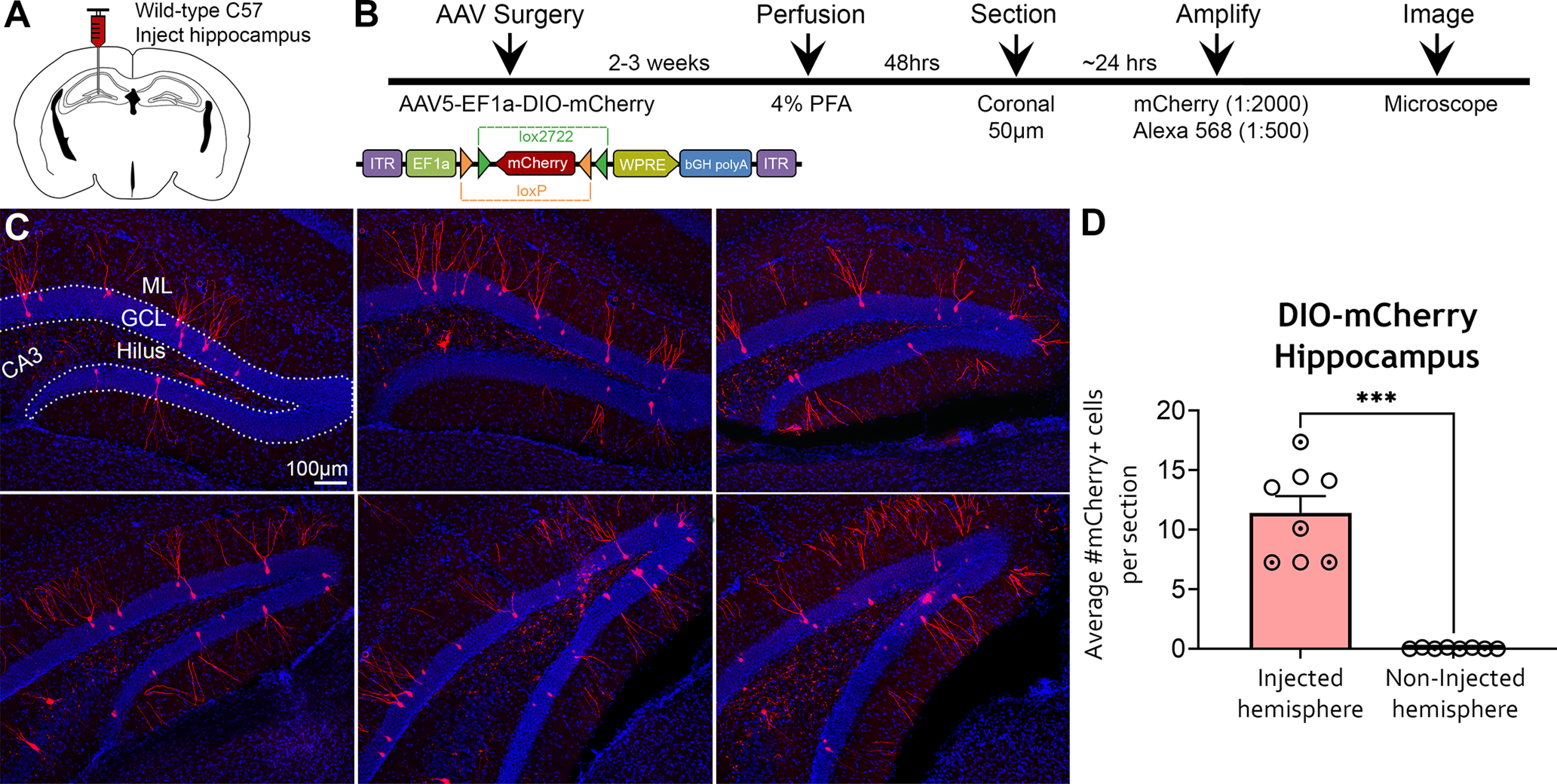
Amplified expression of DIO-mCherry in the hippocampus of WT C57BL/6J mice. ***A***, ***B***, Experimental design and timeline. AAV5-EF1a-DIO-mCherry was injected into the anterior and posterior hippocampi of C57BL/6J mice (*n *=* *8) and perfused 2–3 weeks later. Brains were sectioned in the coronal plane, and viral signal was amplified with rabbit anti-mCherry and goat anti-rabbit 568 antibodies. ***C***, Representative immunofluorescence of mCherry throughout the relatively dorsal (top) and caudal (bottom) DG. Expression of mCherry was primarily observed in the GCL and dendrites extending into the ML (putative dentate GCs). The amplified mCherry signal also resulted in labeling of mossy fibers and cells in the hilus. ***D***, Quantification of mCherry^+^ cells indicated that somatic expression was restricted to the injected hemisphere. Female (clear circles) and male (dotted circles) data points are identified, but no sex differences were found. ***p <* *0.001. Scale bar, 100 μm. Data for this figure are shown in Extended Data Figure 3-1.

To further investigate the off-target expression of AAV5-EF1a-DIO-mCherry in C57BL/6J mice, we quantified the number of mCherry^+^ cells in the anterior and posterior DG following fluorescence signal amplification (*n *=* *8; Fig. 3*A*,*B*). Remarkably, amplified mCherry^+^ cells were found throughout the DG of C57BL/6J mice injected with DIO-mCherry (Fig. 3*C*), almost exclusively restricted to the injected hemisphere (11.43 ± 1.40 cells/section), compared with the noninjected hemisphere (0.05 ± 0.02 cells/section; Mann–Whitney *U *=* *0, *p *<* *0.001; Fig. 3*D*). Importantly, off-target expression was observed in all mice (*n *=* *8; range, 7.25–17.38 cells/section). We also processed a subset of sections with DAB and found that mCherry immunoreactivity was similar to the pattern of amplified DIO-mCherry immunofluorescence (Extended Data Fig. 3-1), indicating that our results were not attributable to nonspecific fluorescence signal. These findings indicate that the off-target expression of DIO constructs in C57BL/6J mice revealed by amplification was highly reproducible.

10.1523/ENEURO.0363-21.2021.f3-1 Figure 3-1 mCherry immunoreactivity in WT C57BL/6J mice injected with AAV5-EF1a-DIO-mCherry. ***A***, Representative photomicrographs of mCherry immunoreactivity in C57BL/6J mice injected with AAV5-EF1a-DIO-mCherry. Overall, mCherry immunoreactivity was comparable to the pattern of expression observed with amplified DIO-mCherry immunofluorescence (Fig. 3). Scale bar, 100 μm. Download Figure 3-1, TIF file.

To determine whether viral titer influenced Cre-independent expression, we performed identical injections in a subset of mice (*n *=* *5), but diluted the AAV5-EF1a-DIO-mCherry construct used in the experiments above by 25% (i.e., 3 μl of stock virus diluted with 1 μl 0.1 m PBS). Mice injected with diluted AAV5-EF1a-DIO-mCherry had ∼75% fewer amplified mCherry^+^ cells (2.52 ± 0.59 cells/section) compared with mice injected with the stock commercial titer (11.43 ± 1.40 cells/section; *t*_(11)_ = 4.785, *p *<* *0.001). These results indicate that high titer viral constructs produce greater off-target expression than diluted viral constructs.

### Fluorescence signal amplification of AAV5-EF1a-DIO-eYFP in WT C57BL/6J mice

**Figure 4.**
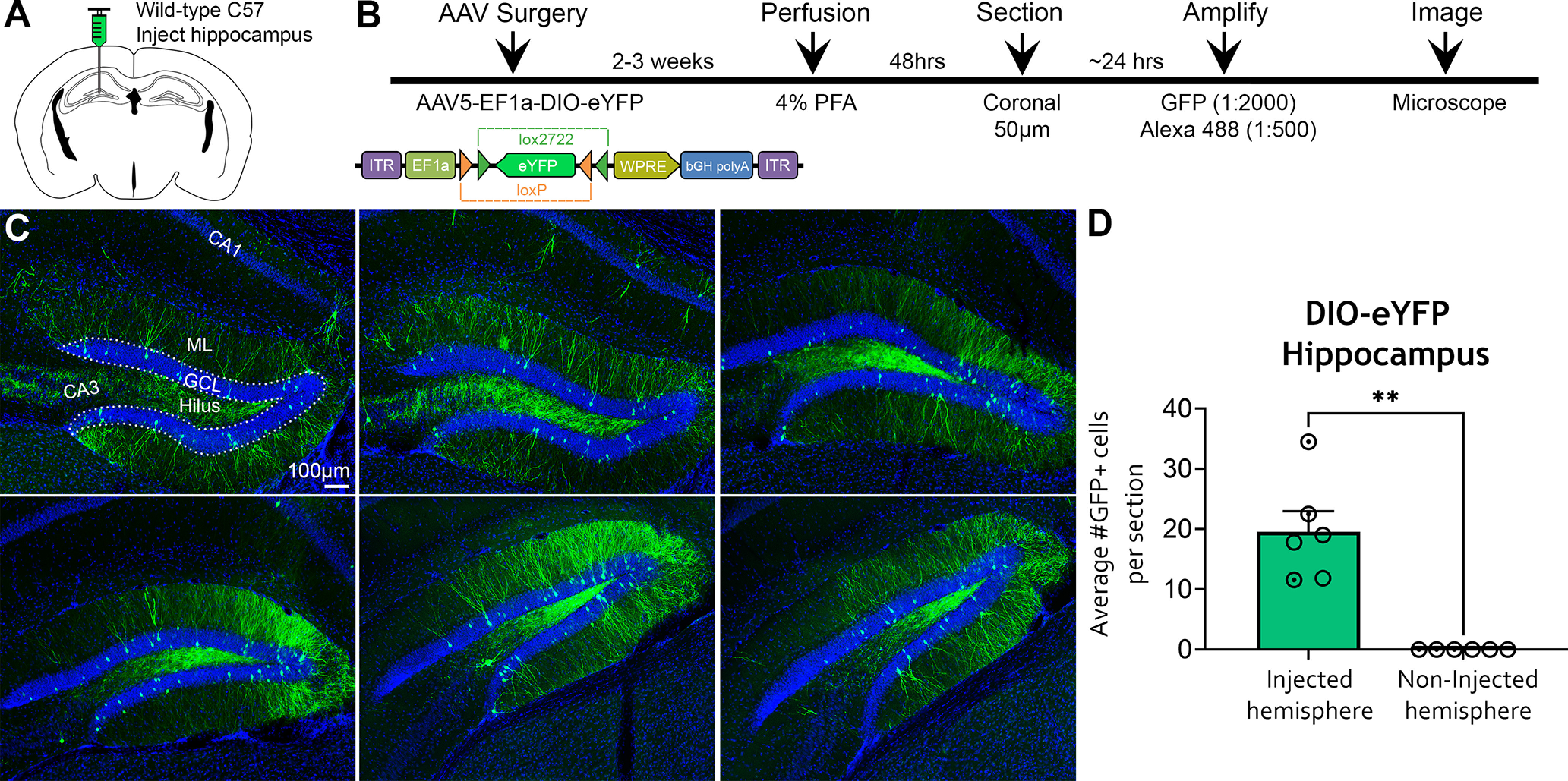
Amplified expression of DIO-eYFP in the hippocampus of WT C57BL/6J mice. ***A***, ***B***, Experimental design and timeline. AAV5-EF1a-DIO-eYFP was injected into the anterior and posterior hippocampi of C57BL/6J mice (*n *=* *6) and perfused 2–3 weeks later. The eYFP signal was amplified with chicken anti-GFP and goat anti-chicken 488 antibodies. ***C***, Representative immunofluorescence of GFP throughout the DG. GFP expression was observed primarily in the DG, characterized by robust labeling of putative GCs within the GCL and their dendrites. The hilus also showed bright GFP signal, with expression in mossy fibers and hilar cells. ***D***, Quantification of GFP^+^ cells revealed that somatic expression was restricted to the injected hemisphere. Female (clear circles) and male (dotted circles) data points are identified, but no sex differences were found. **p <* *0.005. Scale bar, 100 μm. Data for this figure are shown in Extended Data Figures 4-1 and 4-2.

The high expression of amplified DIO-mCherry in C57BL/6J mice prompted us to investigate amplified expression using other DIO constructs. C57BL/6J mice (*n *=* *6) received injections of AAV5-EF1a-DIO-eYFP in the left anterior and posterior DG using identical parameters as the DIO-mCherry experiments (Fig. 4*A*). Mice were euthanized 2–3 weeks after surgery, and brains were sectioned and amplified with anti-GFP antibodies (Fig. 4*B*). Amplification of GFP produced immunofluorescence in the DG that was more extensive than the DIO-mCherry experiments but shared a similar pattern (Fig. 4*C*). Specifically, relatively sparse labeling of GFP^+^ cells was observed in the GCL similar to mCherry amplification. However, amplified GFP^+^ immunofluorescence resulted in robust labeling of dendrites in ML, compared with the relatively sparse labeling of the ML following mCherry amplification (Fig. 4*C*). Furthermore, GFP^+^ immunofluorescence was more pronounced in the hilus, with expression observed in hilar cells and mossy fibers (Fig. 4*C*). As with mCherry, GFP cell counts throughout the DG found that GFP^+^ cells were exclusive to the injected hemisphere (19.54 ± 3.46 cells; noninjected hemisphere, 0.00 ± 0.00 cells; Mann–Whitney *U *=* *0, *p *<* *0.001; Fig. 4*D*). Together, these results demonstrate a highly specific pattern of amplified fluorescence signal of DIO constructs in C57BL/6J mice, independent of the construct used (DIO-mCherry or DIO-eYFP; Extended Data Fig. 4-1).

10.1523/ENEURO.0363-21.2021.f4-1 Figure 4-1 Fluorescence signal amplification of DIO-mCherry and DIO-eYFP is highly specific to the injection site in WT C57BL/6J mice. ***A***, Tile scan of a C57BL/6J mouse injected with AAV5-EF1a-DIO-mCherry. Viral expression was amplified with mCherry antibody. The indent on the top of the left cortex represents a drilling artifact near the injection site. The mCherry expression is primarily restricted to the injected (left) hippocampus, with mCherry^+^ cells observed in the GCL of the DG. There is also sparse labeling of mCherry^+^ cells in the CA3. ***B***, Tile-scan of a C57BL/6J mouse injected with AAV5-EF1a-DIO-eYFP. Viral expression was amplified with GFP and observed primarily within the injected (left) DG. Furthermore, GFP^+^ mossy fiber (MF) axons from dentate GCs were observed projecting to area CA3. Interestingly, commissural GFP^+^ axons, presumably from mossy cells, were observed within the IML of the contralateral hemisphere. Notably, there were no mCherry^+^ or GFP^+^ cells in the noninjected hemisphere. This result indicates that amplified fluorescence signal is highly specific to the target region and the projections of labeled cells. Scale bar, 200 μm. Download Figure 4-1, TIF file.

10.1523/ENEURO.0363-21.2021.f4-2 Figure 4-2 Fluorescence signal amplification in other subfields of the hippocampus. ***A***, ***B***, Viral injections aimed at the DG occasionally resulted in mistargeting, which led to amplified fluorescence signal in other subfields of the hippocampus, such as CA1 or CA2. This finding suggests that amplified viral expression was not unique to the DG, but rather was specific to the injection site. Scale bar, 100 μm. Download Figure 4-2, TIF file.

The amplified expression of mCherry and eYFP in the DG of C57BL/6J mice injected with Cre-dependent constructs led us to question whether off-target expression was unique to the DG or a general consequence of viral injections regardless of the region that was targeted. Serendipitously, we observed amplified immunofluorescence in hippocampal areas CA1 and/or CA2 when viral injections did not target the DG correctly (Extended Data Fig. 4-2).

**Figure 5.**
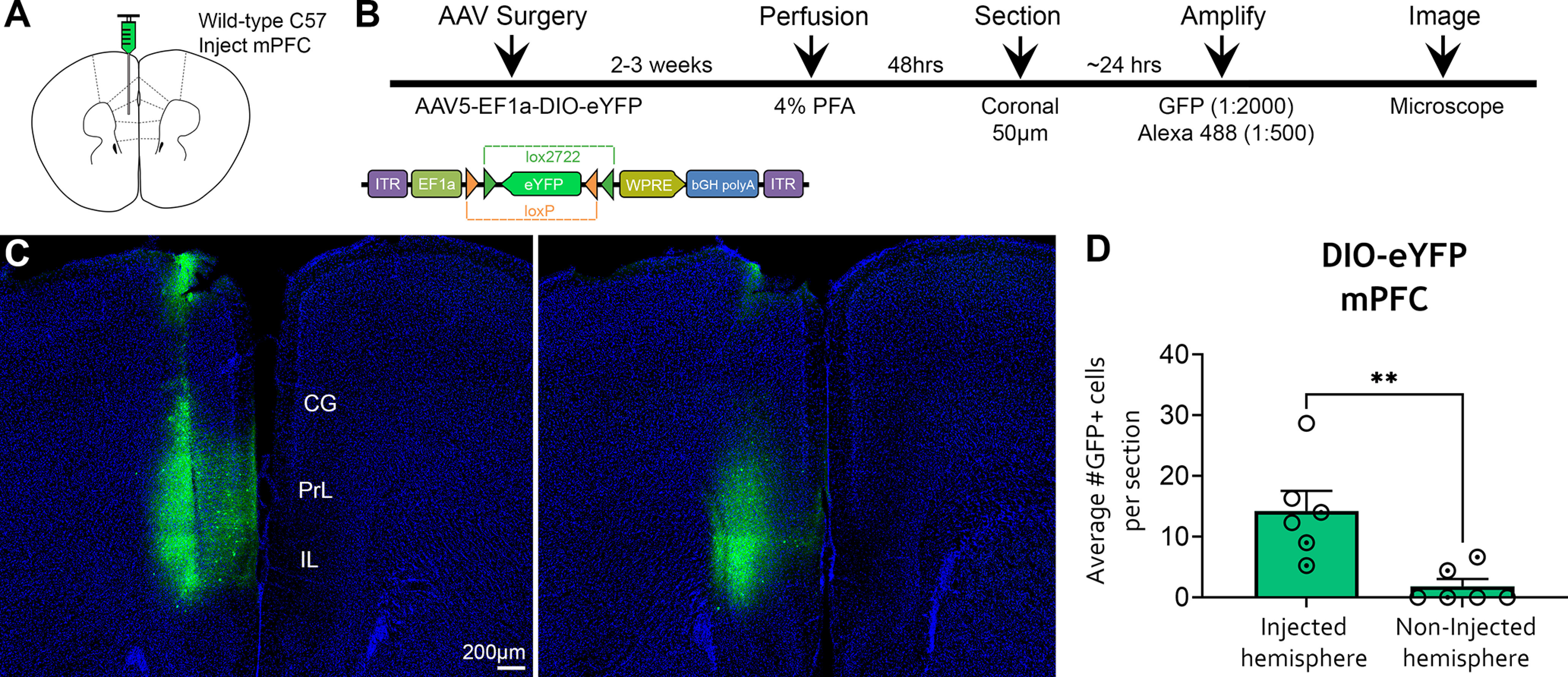
Amplified expression of DIO-eYFP in the mPFC of WT C57BL/6J mice. ***A***, ***B***, Experimental design and timeline. AAV5-EF1a-DIO-eYFP was injected into left mPFC of C57BL/6J mice (*n *=* *6), and mice were perfused 2–3 weeks later. Viral signal was amplified with chicken anti-GFP and goat anti-chicken 488 antibodies. ***C***, Representative GFP immunofluorescence in the mPFC of two sections from the same mouse. ***D***, Quantification of GFP^+^ cells in the mPFC showed that expression was primarily restricted to the injected hemisphere, but two mice had sparse expression of GFP^+^ cells in the noninjected hemisphere, presumably resulting from viral spread because of the close proximity of the left and right mPFC. Female (clear circles) and male (dotted circles) data points are identified, but no sex differences were found. CG, cingulate gyrus; PrL, Prelimbic cortex; IL, infralimbic cortex. **p <* *0.005. Scale bar, 200 μm.

In addition, we specifically targeted the mPFC in C57BL/6J mice (*n *=* *6) using Cre-dependent eYFP (AAV5-EF1a-DIO-eYFP; Fig. 5*A*). The experimental timeline for mPFC experiments was identical to that of eYFP hippocampal injections (Fig. 5*B*). Amplified GFP immunofluorescence was also observed in the mPFC (Fig. 5*C*), at a rate similar to that seen in DG (injected hemisphere, 14.26 ± 3.29 cells/section; vs noninjected hemisphere, 1.84 ± 1.20 cells/section; Mann–Whitney *U *=* *1, *p *=* *0.004; Fig. 5*D*). Overall, these findings suggest that Cre-independent, DIO construct expression is specific to the viral injection site, and is not tied to a particular brain region.

### Fluorescence signal amplification of AAV8-hSyn-DIO-hM3Dq-mCherry in WT C57BL/6J mice

**Figure 6.**
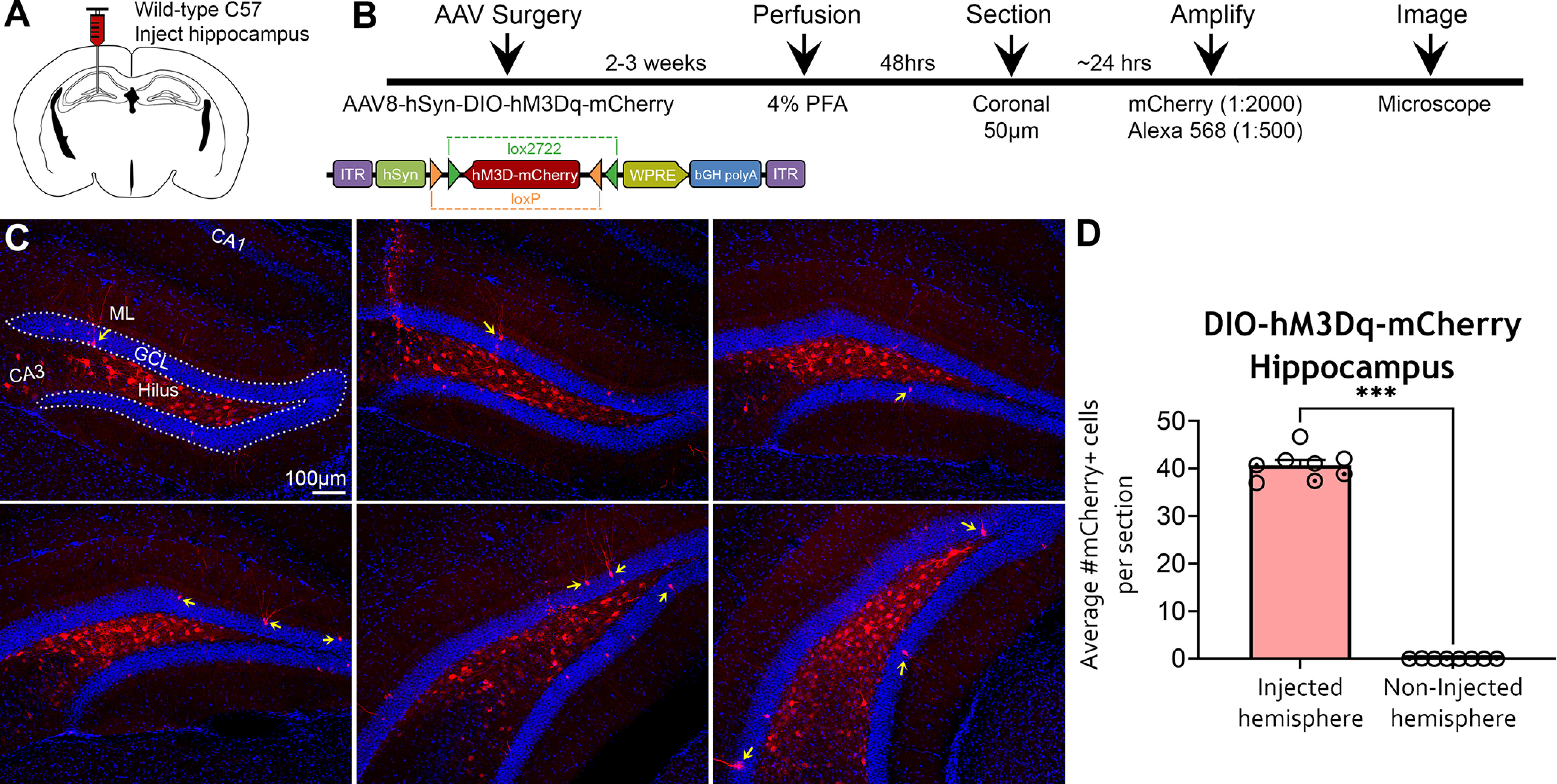
Amplified expression of DIO-hM3Dq-mCherry in the hippocampi of WT C57BL/6J mice. ***A***, ***B***, Experimental design and timeline. AAV8-hSyn-DIO-hM3Dq-mCherry was injected into the anterior and posterior hippocampus of C57BL/6J mice (*n *=* *8), and mice were perfused 2–3 weeks later. The viral signal was amplified with rabbit anti-mCherry and goat anti-rabbit 568 antibodies and was visualized on an epifluorescence microscope. ***C***, Representative mCherry immunofluorescence in relatively dorsal (top) and caudal (bottom) sections of the DG. Amplified mCherry expression appeared primarily within hilar cells and a sparse number of GCs (yellow arrows). ***D***, Quantification of mCherry^+^ cells revealed that expression was restricted to the injected hippocampus. Female (clear circles) and male (dotted circles) data points are identified, but no sex differences were found. ***p <* *0.001. Scale bar, 100 μm. Data for this figure are shown in Extended Data Figures 6-1 and 6-2.

To test whether Cre-independent expression with DIO constructs was restricted to a particular AAV serotype, we used AAV8 Cre-dependent hM3Dq (AAV8-hSyn-DIO-hM3Dq-mCherry). Using the same coordinates as eYFP and mCherry experiments described previously, the DIO-hM3Dq construct was injected into the anterior and posterior DG of C57BL/6 mice (*n *=* *8; Fig. 6*A*). Mice were euthanized 2–3 weeks after surgery, and brain sections were processed for mCherry signal amplification (Fig. 6*B*). Amplification of AAV8-DIO-hM3Dq-mCherry revealed notable fluorescence expression in the DG, indicating that Cre-independent expression was observed across multiple serotypes and promoters. Interestingly, amplification of AAV8-DIO-hM3Dq-mCherry construct revealed a different pattern of fluorescence compared with AAV5-DIO-mCherry (Fig. 6*C*). Specifically, AAV8-hSyn-DIO-hM3Dq mCherry^+^ immunofluorescence was primarily observed in hilar neurons, with some sparse labeling in GCs specific to the injected hemisphere (40.73 ± 1.09 cells; vs noninjected hemisphere, 0.01 ± 0.01 cells; Mann–Whitney *U *=* *0, *p *<* *0.001; Fig. 6*D*). Notably, the AAV8-hSyn-DIO-hM3Dq-mCherry construct differed from the previous constructs we tested in two ways: serotype (AAV8) and promoter (hSyn; as opposed to EF1a in previous experiments). A subset of sections processed with DAB revealed that mCherry immunoreactivity under the hSyn promoter matched the pattern of amplified DIO-hM3Dq-mCherry immunofluorescence (Extended Data Fig. 6-1).

10.1523/ENEURO.0363-21.2021.f6-1 Figure 6-1 mCherry immunoreactivity in WT C57BL/6J mice injected with AAV8-hSyn-DIO-hM3Dq-mCherry. ***A***, Representative photomicrographs of mCherry immunoreactivity in C57BL/6J mice injected with AAV8-hSyn-DIO-hM3Dq-mCherry. The pattern of mCherry immunoreactivity was comparable to the amplified immunofluorescence of DIO-hM3Dq-mCherry (Fig. 6). Scale bar, 100 μm. Download Figure 6-1, TIF file.

10.1523/ENEURO.0363-21.2021.f6-2 Figure 6-2 Fluorescence signal amplification of AAV5-hSyn-DIO-hM4Di-mCherry in WT C57BL/6J mice. ***A***, C57BL/6J mice were injected in the DG with AAV5-hSyn-DIO-hM4D(Gi)-mCherry, and sections were amplified with mCherry. Interestingly, mCherry^+^ cells were primarily located in the hilus, but a small number of GCs were also labeled. The pattern of amplified AAV5-hSyn-DIO-hM4Di-mCherry expression is consistent with the AAV8-hSyn-DIO-hM3Dq-mCherry construct shown in Figure 6. Scale bar, 100 μm. Download Figure 6-2, TIF file.

To determine whether the expression difference was because of serotype, we injected AAV5-hSyn-DIO-hM4Di-mCherry into C57BL/6J mice. We found that mCherry amplification of AAV5-hSyn-DIO-hM4Di-mCherry had a pattern of fluorescence similar to that of AAV8-hSyn-DIO-hM3Dq-mCherry, indicating that serotype is not driving the difference in the pattern of Cre-independent expression (Extended Data Fig. 6-2). These results suggest that DIO constructs with the EF1a and hSyn promoters may show preferential expression in GCs versus hilar cells, respectively, in C57BL/6J mice. Moreover, these results also demonstrate that off-target expression of DIO constructs was observed using constructs from different vendors (UNC Core, Addgene).

### Contextual fear learning and memory

**Figure 7.**
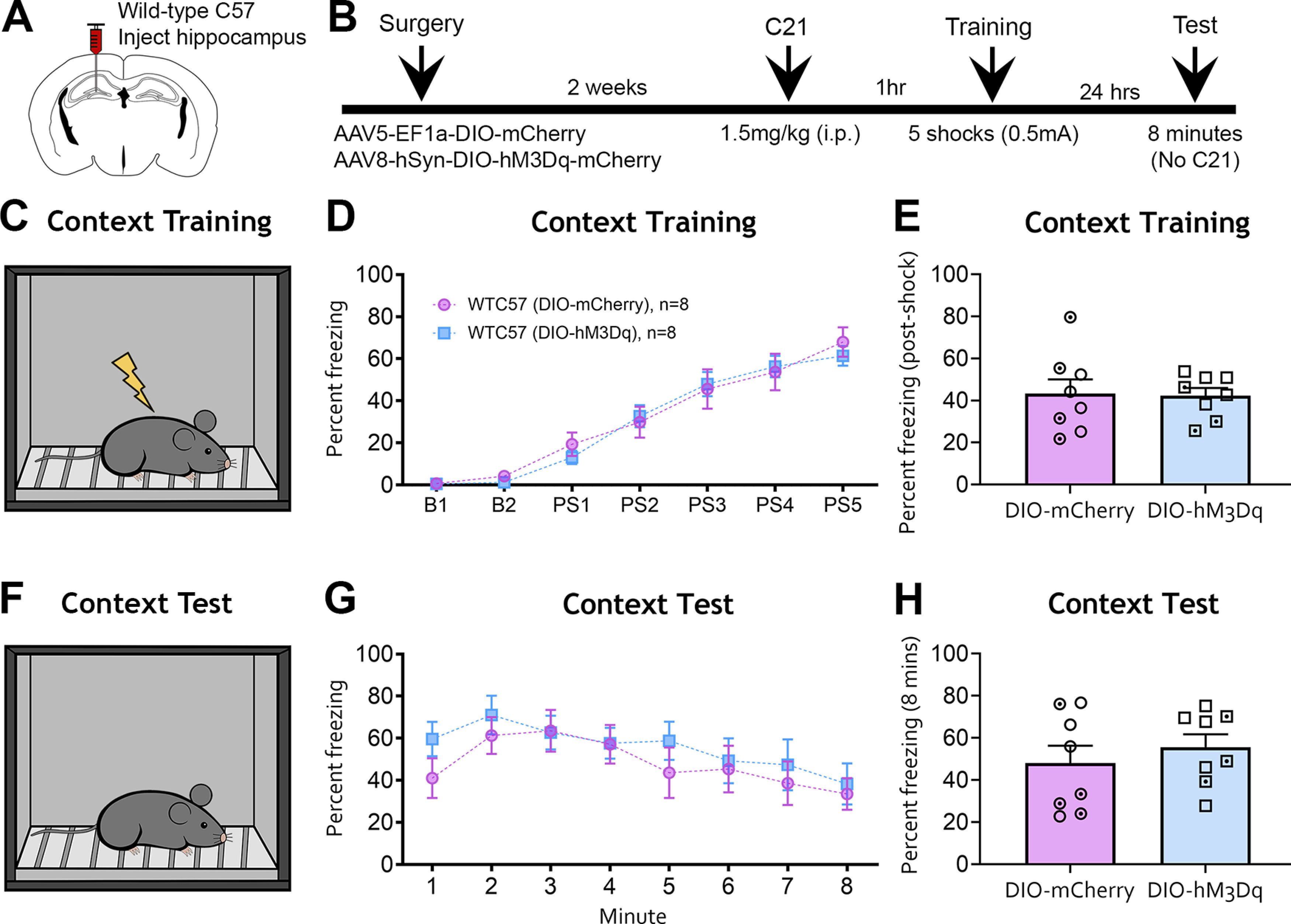
The hM3Dq agonist C21 does not affect fear behavior in C57BL/6J mice injected with DIO-mCherry or DIO-hM3Dq-mCherry in the DG. ***A***, ***B***, Experimental design and timeline. Adult C57BL/6J mice underwent surgery to receive intrahippocampal injections of AAV-EF1a-DIO-mCherry or AAV-hSyn-DIO-hM3Dq-mCherry. After a 2 week recovery period, mice were injected with the hM3Dq agonist C21 1 h before contextual fear training. ***C***, Mice were then placed in a fear-conditioning chamber. Baseline activity was assessed over 2 min, followed by five footshocks (0.5 mA) spaced 1 min apart. ***D***, Minute-by-minute analysis of the training session revealed that freezing behavior did not differ between EF1a-DIO-mCherry or hSyn-DIO-hM3Dq-mCherry groups. ***E***, The average postshock freezing did not differ between the EF1a-DIO-mCherry and hSyn-DIO-hM3Dq-mCherry groups. ***F***, Mice were returned to the same operant chamber 24 h later to test contextual fear memory. Notably, C21 was not administered a second time before the contextual memory test. ***G***, Minute-by-minute analysis revealed that conditioned freezing did not differ between the EF1a-DIO-mCherry or hSyn-DIO-hM3Dq-mCherry groups. ***H***, Average freezing during the memory test did not differ between groups. Female (clear points) and male (dotted points) data points are identified, but no sex differences were found.

Next, we sought to determine whether the off-target expression of Cre-dependent viral constructs in C57BL/6J mice could influence behavior. Given the number of DIO-hM3Dq-mCherry cells observed in the hilus after fluorescence signal amplification (Fig. 6), and a recent study that reported chemogenetic excitation of hilar cells impaired contextual fear learning and memory (Botterill et al., 2021), we were curious whether similar impairments would be observed in control mice injected with the DIO construct. Adult C57BL/6J mice were injected in the anterior and posterior DG with AAV5-EF1a-DIO-mCherry or AAV8-hSyn-DIO-hM3Dq-mCherry (*n *=* *8/group; Fig. 7*A*). After a 2 week postsurgical recovery period, mice were injected with the hM3Dq agonist C21 (1.5 mg/kg, i.p.) 1 h before contextual fear training (Fig. 7*B*,*C*).

C21 treatment before contextual fear training had no effect on freezing behavior during training in mice injected with DIO-hM3Dq-mCherry versus DIO-mCherry (two-way repeated-measures ANOVA: *F*_(1,14)_ = 0.045, *p *=* *0.834; Fig. 7*D*). The two-way repeated-measures ANOVA also revealed a significant main effect of time (*F*_(6,84)_ = 72.69, *p *<* *0.001), attributable to increased freezing behavior as the task progressed from baseline freezing to postshock periods. However, there was no significant interaction between treatment and time (*F*_(6,84)_ = 0.474, *p *=* *0.825). When postshock freezing was averaged across all five postshock periods, there was no difference in freezing behavior between mice injected with DIO-mCherry (43.32 ± 6.70%) or DIO-hM3Dq-mCherry (42.30 ± 3.63%; unpaired *t* test: *t*_(14)_ = 0.133, *p *=* *0.895; Fig. 7*E*). Together, these results showed no detectable behavioral effect of the hM3Dq agonist C21 in C57BL/6J mice injected with DIO-hM3Dq-mCherry.

To evaluate contextual fear memory retrieval, mice were returned to the same fear-conditioning chamber 24 h after training, and freezing behavior was evaluated over 8 min (Fig. 7*F*). Importantly, C21 was not given before the memory test. There was no difference in memory retrieval between the DIO-mCherry and DIO-hM3Dq-mCherry groups (two-way repeated-measures ANOVA: *F*_(1,14)_ = 0.542, *p *=* *0.474; Fig. 7*G*). However, the two-way repeated-measures ANOVA found a significant main effect of time (*F*_(7,98)_ = 4.483, *p *<* *0.001), which was attributable to a gradual decline in freezing behavior over the duration of the test. There was no interaction between treatment and time (*F*_(7,98)_ = 0.512, *p *=* *0.824). Average freezing behavior over the entire session also did not differ between DIO-mCherry (48.02 ± 8.24%) and DIO-hM3Dq-mCherry groups (55.60 ± 6.15%; unpaired *t* test: *t*_(14)_ = 0.737, *p *=* *0.474; Fig. 7*H*). Collectively, these results suggest that the hM3Dq agonist C21 did not influence fear learning or memory retrieval in C57BL/6J mice injected with DIO-hM3Dq-mCherry. Further increasing the number of hippocampal DIO-hM3Dq-mCherry cells via bilateral injections (i.e., four injections total) also had no effect on fear learning or memory (all *p* values > 0.166). These results indicate that C21 had no effect on fear behavior in C57BL/6J mice injected with DIO-hM3Dq-mCherry.

### mCherry and c-Fos immunofluorescence following C21 challenge

**Figure 8.**
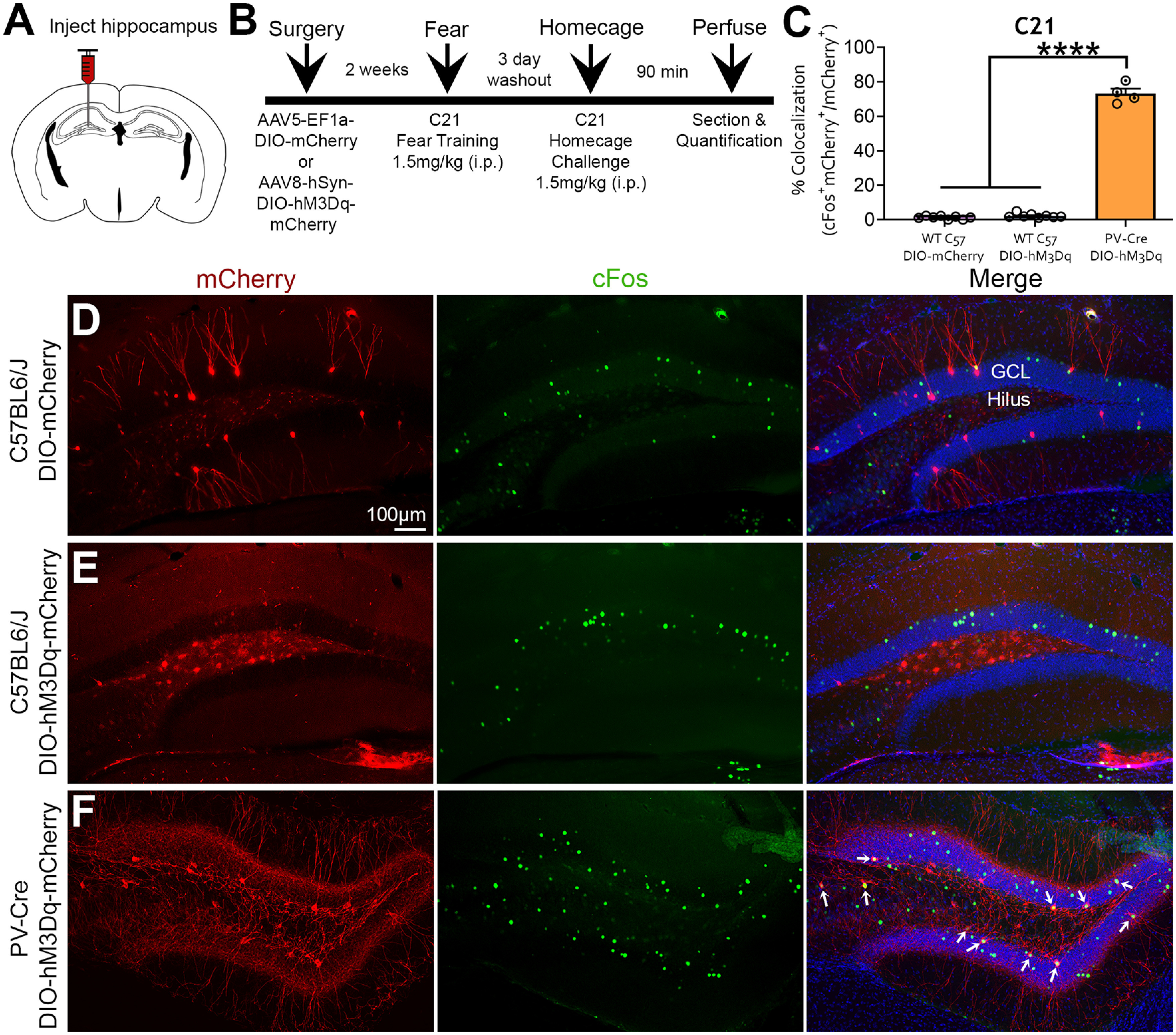
mCherry and c-Fos immunofluorescence following C21 home-cage challenge. ***A***, ***B***, Experimental design and timeline. Mice underwent surgery for AAV injection and were allowed 2 weeks for recovery. Mice underwent behavioral testing and were then given a 3 d washout period. Mice were then injected with C21 (1.5 mg/kg) in their home cage and were euthanized 90 min later to evaluate the immediate early gene c-Fos. ***C***, The percentage of colocalization of c-Fos^+^ and mCherry^+^ cells following C21 challenge was significantly lower in C57BL/6J mice injected with DIO-mCherry (7 c-Fos^+^mCherry^+^/497 mCherry^+^ cells = 1.41%) or DIO-hM3Dq-mCherry (23 c-Fos^+^mCherry^+^/1062 mCherry^+^ cells = 2.17%) compared with *PV-Cre*^+^ mice injected with DIO-hM3Dq-mCherry (267 c-Fos^+^mCherry/367 mCherry^+^ cells = 72.75%). ***D–F***, Representative images show that C57BL/6J mice lacked the clear elevation of c-Fos (green) in mCherry^+^ cells seen in *PV-Cre*^+^ mice (yellow; white arrows). ****p <* *0.0001. Scale bar, 100 μm.

Despite observing no behavioral effect of C21 in the DIO-hM3Dq-mCherry group, we wanted to determine whether C21 could activate DIO-hM3Dq-mCherry^+^ neurons in C57BL/6J mice by evaluating the immediate early gene c-Fos. Mice were given a 3 d washout period after fear memory retrieval and then injected with C21 (1.5 mg/kg, i.p.) in their home cage and euthanized 90 min later (Fig. 8*A*,*B*). Next, we quantified the percentage of colocalization of c-Fos and mCherry cells by dividing the number of c-Fos^+^mCherry^+^ cells by the total number of mCherry^+^ cells. A one-way ANOVA revealed a significant main effect of treatment (*F*_(2,17)_ = 1211, *p *<* *0.0001; Fig. 8*C*). Tukey’s *post hoc* test indicated that the number of colocalized c-Fos^+^mCherry^+^ cells was significantly greater in the *PV-Cre*^+^ mice injected with DIO-hM3Dq-mCherry (73.20 ± 2.86% of cells) than C57BL/6J mice injected with DIO-mCherry (1.26 ± 0.31% of cells) and DIO-hM3Dq-mCherry (2.27 ± 0.44% of cells; all *p* values < 0.0001; Fig. 8*C*). Importantly, colocalization of c-Fos^+^ and mCherry^+^ cells did not differ in C57BL6/J mice injected with DIO-mCherry versus DIO-hM3Dq-mCherry (*p *=* *0.719). Together, these results confirm that the hM3Dq agonist C21 potently activates DIO-hM3Dq-mCherry^+^ neurons in Cre^+^ mice, an effect that is absent in WT C57BL/6J mice (Fig. 8).

## Discussion

The present study investigated anatomic and behavioral effects of Cre-dependent rAAVs in mice lacking Cre-recombinase. WT C57BL/6J mice injected with Cre-dependent viral constructs showed minimal nonamplified fluorescence, consistent with the notion that “leak” expression is a rare phenomenon in DIO constructs (Fenno et al., 2011). However, antibody amplification of the fluorescent reporter proteins eYFP or mCherry revealed fluorescence in different brain regions where virus was injected. Subsequent experiments failed to show any behavioral or immediate early gene effect of DIO-hM3Dq-mCherry in C57BL/6J mice injected with the hM3Dq agonist C21. These results suggest that Cre-dependent rAAVs injected in mice lacking Cre can result in off-target transgene expression, as revealed by fluorescence signal after antibody amplification, but without yielding notable behavioral or functional effects in our experimental system.

### Fluorescence signal amplification of viral expression

In this work we evaluated fluorescence signal amplification in Cre^+^ and C57BL/6J mice injected with various Cre-dependent rAAVs. First, we evaluated *TH-Cre*^+^ mice injected with DIO-mCherry and found that the expression of fluorescently labeled cell bodies in the VTA were consistent with previous studies (Stuber et al., 2010; Mahler et al., 2019). However, nonamplified fluorescence of VTA projections into the NAc-DS or mPFC were notably weak, and fluorescence signal amplification improved the visualization of mCherry, especially in mPFC axon terminals (Fig. 1*C*). We also evaluated *PV-Cre*^+^ mice injected with DIO-mCherry or DIO-eYFP in the DG and found that while nonamplified fluorescence was suitable for visualizing PV^+^ cells, fluorescence signal amplification improved expression in fine processes such as dendrites. Collectively, these findings support the notion that fluorescence signal amplification can significantly improve visualization of viral transgene expression (Falcy et al., 2020).

We also tested the specificity of Cre-dependent rAAVs in C57BL/6J mice. We observed minimal nonamplified fluorescence, consistent with the dependence of Cre-recombinase to drive transgene expression (Fenno et al., 2011; Fischer et al., 2019). However, we found that fluorescence signal amplification reliably labeled mCherry^+^ or GFP^+^ cells wherever the Cre-dependent rAAV was injected (e.g., DG, CA1, or mPFC). Importantly, there were few or no amplified cells in the noninjected hemisphere, suggesting that antibody specificity was not an issue. Furthermore, fluorescence amplification revealed substantial AAV-DIO expression in C57BL/6J mice regardless of the commercial vendor (Addgene, UNC Core), serotype (AAV5, AAV8), or promoter (EF1a, hSyn) used. These observations indicate that our results could apply to a broad range of rAAV DIO constructs. Overall, these findings warrant caution in interpreting the results of DIO constructs in Cre^–^ subjects, especially if quantitative measures are used following fluorescence signal amplification.

### Functional considerations

Upon discovering the effect of fluorescence signal amplification in C57BL/6J mice injected with Cre-dependent rAAVs, we considered the implications for off-target effects in mice typically assigned as controls. We used the hM3Dq agonist C21 to determine whether the expression of DIO-hM3Dq in C57BL/6J mice had any functional effects. We found that C21 had no impact on contextual fear learning or memory retrieval and was insufficient to trigger a clear elevation of c-Fos expression in DIO-hM3Dq-mCherry^+^ cells of C57BL/6J mice, as was observed in *PV-Cre*^+^ mice. Our results are consistent with previous studies that found no effect of DIO-hM3Dq in Cre^–^ subjects injected with DREADD agonists compared with Cre^+^ counterparts (Alexander et al., 2018; Bonaventura et al., 2019; Mahler et al., 2019), suggesting that DIO construct expression levels in C57BL/6J mice may be insufficient to modulate neuronal activity and affect behavior. Nevertheless, expression level thresholds for phenotypic change will differ between experimental contexts, and as such it cannot be ruled out that functional consequences can arise from off-target gene expression from Cre-dependent rAAV.

### Technical considerations

#### Viral titer and injection volume

Specificity of viral expression is a common concern in experiments that use rAAVs. Viral titer and injection volume represent two main factors that can impact viral expression, and thereby might modulate DIO-construct expression in Cre^–^ animals. High-titer rAAVs are required to introduce numerous viral particles within a single cell to achieve adequate viral expression. For neuroscience applications, commercial vendors typically provide rAAVs at titers ranging between ≥1 × 10^11^ and ∼10^13^ vg/ml. However, the relationship between vector dose and protein expression is nonlinear. For example, a study reported a sixfold increase in the number of virally labeled cells when viral titer was adjusted from 5 × 10^12^ to 5 × 10^13^ vg/ml (Zingg et al., 2017). A second factor to consider is viral injection volume, which is often influenced by factors such as experimental design or the size of the brain region that is targeted. For large brain regions like the hippocampus, injection volumes of ∼0.25 μl are relatively common, but numerous studies have injected volumes ≥0.5 μl and reported good specificity (Gundersen et al., 2013; Bui et al., 2018; Piatkevich et al., 2019; Johnston et al., 2021).

In the present study, stock rAAV titers from commercial vendors (≥4 × 10^12^ vg/ml) were used at relatively low injection volumes (0.2 μl) because these parameters achieved highly specific expression in *TH-Cre*^+^ and *PV-Cre*^+^ mice. In C57BL/6J mice, this injection volume yielded minimal nonamplified fluorescence, but increased immunofluorescence following antibody amplification. The off-target expression of DIO constructs observed with amplification was reduced by ∼75% when viral constructs were diluted by 25%. These results suggest that dilution of viral titer is a possible mitigation strategy to minimize off-target rAAV expression; however, titer reduction could potentially have a negative impact on experimental outcomes by missing phenotypes that are only observable with robust transgene expression.

#### Causes of off-target expression in mice lacking Cre

The cause of off-target Cre-independent rAAV transgene expression was not investigated within the scope of this study. Spontaneous reversion of DIO constructs is known to occur at a low rate and is likely to be the origin of some of this expression. In support of this, a previous study evaluated recombinant plasmids and found that between 1 in 1000 and 1 in 10,000 copies contained a reverted transgene (Fischer et al., 2019).

However, given our detection of substantial numbers of low-intensity transgene-expressing cells, we suspect that there are factors additional to transgene reversion that could result in Cre-independent expression of DIO constructs. The ITRs of AAV are known to exhibit transcriptional activity in a number of cell types, with the AAV2 ITRs, which are used in the majority of applications, exhibiting stronger promoter activity than ITRs from several other serotypes (Earley et al., 2020). Indeed, early rAAV gene therapy constructs for cystic fibrosis relied on this activity to drive expression of the CFTR (cystic fibrosis transmembrane conductance regulator) gene (Flotte et al., 1992). It is possible that in C57BL/6J mice, weak expression of the transgene could be achieved through transcriptional activity of the ITR, although transcriptional activity of ITRs is yet to be directly tested in neuronal cell populations.

Furthermore, within the nucleus, rAAV largely exists in a concatemeric, episomal state (Yang et al., 1999). Where the head is the 5′ end of the rAAV genome and the tail is the 3′ end, the configurations of multiple rAAV genomes can be head-to-head, head-to-tail, or tail-to-tail. If multiple copies of nonreverted DIO constructs were present within a single cell, it is possible that in the tail-to-tail configuration, promoter activity from one DIO genome could read through the rAAV sequence to translate the encoded protein in a second genome of the concatemer. Indeed, this reliance on transcription across multiple genomes is used to yield expression from large gene constructs, using splice donor and acceptor sites in the two respective rAAV genomes (Trapani et al., 2015).

Finally, while AAV is largely considered to be a nonintegrating vector, it is known that integration events do occur at low levels. It is possible that if the DIO construct integrated at a transcriptionally active locus, translation of the nonreverted transgene could be initiated. Indeed, this is the basis on which promoterless rAAV constructs for rAAV-mediated gene therapy operate, albeit in a more actively targeted and efficient manner (Barzel et al., 2015).

#### Minimizing off-target expression in DIO constructs

A previous study revealed that both loxP site mutation and decoupling the start codon from the gene to a position outside of the loxP inversion sites were required to achieve dramatic reduction in off-target expression from DIO/FLEX rAAV constructs, a system referred to as “ATG-out” (Fischer et al., 2019). This suggests that transgene reversion is not the only cause of off-target expression in neurons following DIO construct delivery, because, if this was the case, loxP mutation alone would have been sufficient to minimize this effect. At present, this strategy has not been widely implemented in the neuroscience field but should be considered by those using sensitive systems and/or cell counting assays. Importantly, the ATG-out system, while vastly reducing off-target activity, did not entirely abrogate expression in the system, and was not assessed within the context of signal amplification. Further work should be performed to ensure the fidelity of ATG-out vectors in signal-amplified samples and to explore other approaches for improving the specificity of inducible transgene systems for use in neuroscience applications.

#### Specificity of Cre-recombinase

Cre-dependent rAAVs are generally considered to have a high degree of specificity because of the dependance of Cre-recombinase to drive transgene expression (Huang et al., 2014; Saunders and Sabatini, 2015; McLellan et al., 2017; Haggerty et al., 2020). However, specificity of Cre-recombinase can be influenced by factors such as breeding, genotyping, and/or germline recombination (Song and Palmiter, 2018). Specificity problems are particularly well documented in tamoxifen-inducible transgenic lines (Stifter and Greter, 2020; Van Hove et al., 2020). Therefore, it is important to consider the specificity of transgenic lines in addition to rAAV titer and injection volume.

#### Implications for control experiments

Selecting appropriate controls is a critical step in designing rAAV experiments, especially for studies that involve cell and/or circuit manipulations. There are several strategies for rAAV controls, and each approach has strengths and weaknesses. For example, a popular strategy involves injecting Cre^+^ mice with identical rAAV constructs and randomly assigning subjects to a treatment [e.g., clozapine *N*-oxide (CNO) or C21] or control group (e.g., saline). Although this strategy controls for genotype and viral construct, it often overlooks the effect of treatment. Indeed, compounds such as CNO can have off-target effects (MacLaren et al., 2016; Gomez et al., 2017; Manvich et al., 2018), and therefore these experiments often require additional controls that receive treatment but not the same rAAV construct. A second strategy involves comparing Cre^+^ to Cre^–^ littermates injected with identical rAAV constructs (Smith et al., 2016). This strategy offers the benefit of treating all subjects identically but does not account for potential genotype effects in Cre^+^ mice. Moreover, this strategy requires additional steps such as confirmation of genotypes and/or evaluation of viral expression in Cre^+^ versus Cre^–^ mice. Last, another popular strategy involves injecting Cre^+^ mice with gain-of-function or loss-of function rAAV constructs, and control mice with an rAAV construct that only encodes a fluorescent protein such as mCherry or eYFP. This strategy also allows for all mice to receive the same treatment (e.g., CNO or light pulses). This approach is widely used because of the low risk of off-target effects in control mice, but the disadvantage is the use of different viral constructs.

Although we did not observe any functional off-target effects of Cre-dependent rAAVs in the DG of C57BL/6J mice, we did not evaluate factors such as different behavioral tasks, greater rAAV injection volumes (e.g., 0.5 μl), rAAV injections in different brain regions, or higher doses of C21. Based on the results of the current study, we suggest caution when choosing controls for gain-of-function or loss-of function Cre-dependent constructs. Our data point to the use of fluorophore-only controls as the preferential option to minimize potential off-target effects of Cre-dependent rAAV constructs in control mice.

### Conclusions

Cre-recombinase-dependent rAAVs represent a powerful tool that many neuroscientists use for labeling, tracing, or manipulating specific neuronal populations. Although the fluorescent reporter of most viral constructs yields suitable transgene expression levels within infected cell populations, many laboratories use antibody-based fluorescence signal amplification to visualize weak or intermediate fluorescence signals. Here, we report the observation that Cre-dependent AAVs injected into different brain regions of mice lacking Cre-recombinase reliably showed expression following antibody amplification of the fluorescent reporter. Our results therefore caution that researchers must carefully design and interpret data involving Cre-dependent rAAV infection.

**Table 1:** rAAV information: summary table of the Cre-dependent rAAVs used in the present study

Construct	Serotype	Titer	Injection volume	Vendor	Figures
AAV-EF1a-DIO-eYFP	5	≥4 × 10^12^ vg/ml	0.2 μl/site	UNC Core	1, 2, 4, 5, Extended Data 4–1, Extended Data 4–2
AAV-EF1a-DIO-mCherry	5	≥7 × 10^12^ vg/ml	0.2 μl/site	UNC Core	1, 2, 3, 8, 3–1, Extended Data 4–1, Extended Data 4–2
AAV-hSyn-DIO-hM3D(Gq)- mCherry	8	≥5 × 10^12^ vg/ml	0.2 μl/site	UNC Core	6, 8, Extended Data 6–1
AAV-hSyn-DIO-hM4D(Gi)- mCherry	5	≥8 × 10^12^ vg/ml	0.2 μl/site	Addgene (catalog #44362)	Extended Data 6–2

Overall, four Cre-dependent constructs were used that differed by promoter (EF1a, hSyn), serotype (AAV5, AAV8), and/or vendor (UNC Core, Addgene). High-titer (≥4 × 10^12^ vg/ml) stock virus (0.2 μl) was injected into each region of interest.

**Table 2:** Antibody information with details of the primary and secondary antibodies used in the present study

Antigen	Host	Description	Dilution	Catalog #	Vendor	RRID#
GFP	Chicken	Polyclonal	1:2000	AB13970	Abcam	AB_300798
mCherry	Rabbit	Polyclonal	1:2000	167453	Abcam	AB_2571870
mCherry	Rat	Monoclonal	1:1000	M11217	Thermo Fisher Scientific	AB_2536611
c-Fos	Rabbit	Polyclonal	1:2500	226003	Synaptic Systems	AB_2231974
Anti-rabbit (HRP conjugate)	Donkey	Polyclonal	1:500	711-036-152	Jackson ImmunoResearch	AB_2340590
Anti-rabbit	Goat	Biotinylated IgG	1:500	BA-1000	Vector Laboratories	AB_2313606
Alexa Fluor 488 anti-chicken	Goat	Fluorescence (488 nm)	1:500	A-11039	Thermo Fisher Scientific	AB_2534096
Alexa Fluor 568 anti-rabbit	Goat	Fluorescence (568 nm)	1:500	A-11011	Thermo Fisher Scientific	AB_143157

## Synthesis

Reviewing Editor: Arianna Maffei, SUNY Stony Brook Decisions are customarily a result of the Reviewing Editor and the peer reviewers coming together and discussing their recommendations until a consensus is reached. When revisions are invited, a fact-based synthesis statement explaining their decision and outlining what is needed to prepare a revision will be listed below. The following reviewer(s) agreed to reveal their identity: Jason Snyder, Michael Drew.

There is strong agreement between the reviewers that this is an interesting and well executed study. The reviewers had some minor concerns that are outlines below

Reviewer #1

This is a straightforward study that investigated the extent of non-specific gene expression following injection of DIO AAVs. They show that off target expression is consistent, occurs in many brain regions, using different immuno-visualization methods, and with different serotypes of AAVs. The authors were able to show this largely because of signal amplification, which allowed for visualization of fluorescent proteins that were otherwise weak and likely ignored in previous studies. This is an important study that will be of broad interest. I have few suggestions for improvement:

It could be valuable for Fig 8 to have quantitative data to substantiate claims of minimal fos expression in mcherry and hm3d mice. Or even just images showing minimal (as opposed to zero) expression. Alternatively, the claims could be toned down (eg “we did not observe obvious elevation in fos expression in mcherry cells...”. Additionally, it looks like some of the fos-positive cells in the DG have weak mcherry signal (eg cluster of 3-4 cells in the middle of the suprapyramidal blade). Maybe this is due to bleedthrough; likely not a problem.

Minor:

Abstract: “hippocampus and cortex", but hippocampus is (a part of) cortex. Maybe say neocortex.

Reviewer #2:

Overall this is an interesting paper. Initially I was skeptical that such leaky expression could occur, particularly given the construction of the DIO vectors. However, I find the experiments convincing and the discussion section nicely describes some potential mechanisms.

I also appreciate that the authors conducted the functional studies with the DREADDs and report no obvious effects on behavior or IEG expression. This is important, as it suggests that the many studies employing AAV-DIO constructs may still be valid. It is important to recognize that the authors have chosen an assay (contextual fear conditioning) that should be quite sensitive to increased neural activity caused by the Gq DREADD (e.g., Krueger et al., 2020 and unpublished data from my lab). So the null effect is meaningful.

Given the lack of functional effects, I think it would be appropriate to adjust the wording of sections of the paper. E.g., the intro says “our results indicate that DIO constructs have considerable off-target expression in wild type subjects.” The discussion says something similar. According to my dictionary “considerable” means “notably large in size.” I don’t think the leaky expression is notably large. Furthermore, this wording is subjective. I suggest the word “considerable” and any other such adjectives describing the effect size be removed.

My only other suggestion is that the authors create a table displaying the source and description of the AAV constructs used in each figure. This information is already in the paper, but it would be helpful to have it immediately at hand while one is reviewing the figures.
