# Supplementary figures and images for: Off-Target Expression of Cre-Dependent Adeno-Associated Viruses in Wild-Type C57BL/6J Mice
Source: eNeuro. 2021 Nov 24;8(6):ENEURO.0363-21.2021. doi: 10.1523/ENEURO.0363-21.2021 (PMC8614227; doi:10.1523/ENEURO.0363-21.2021)

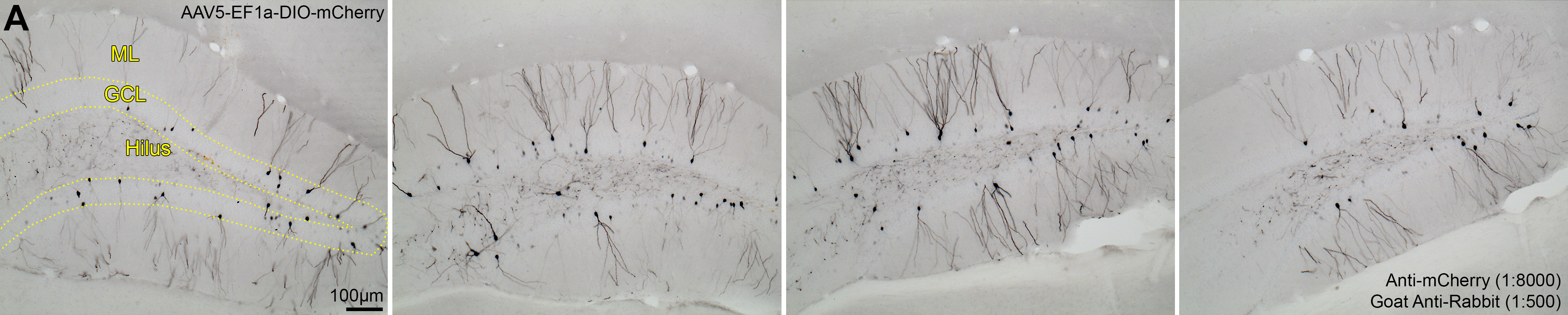

Supplement: Figure 3-1 — mCherry immunoreactivity in WT C57BL/6J mice injected with AAV5-EF1a-DIO-mCherry. A, Representative photomicrographs of mCherry immunoreactivity in C57BL/6J mice injected with AAV5-EF1a-DIO-mCherry. Overall, mCherry immunoreactivity was comparable to the pattern of expression observed with amplified DIO-mCherry immunofluorescence (Fig. 3). Scale bar, 100 μm. Download Figure 3-1, TIF file. [file enu-eN-NWR-0363-21-s02.tif]

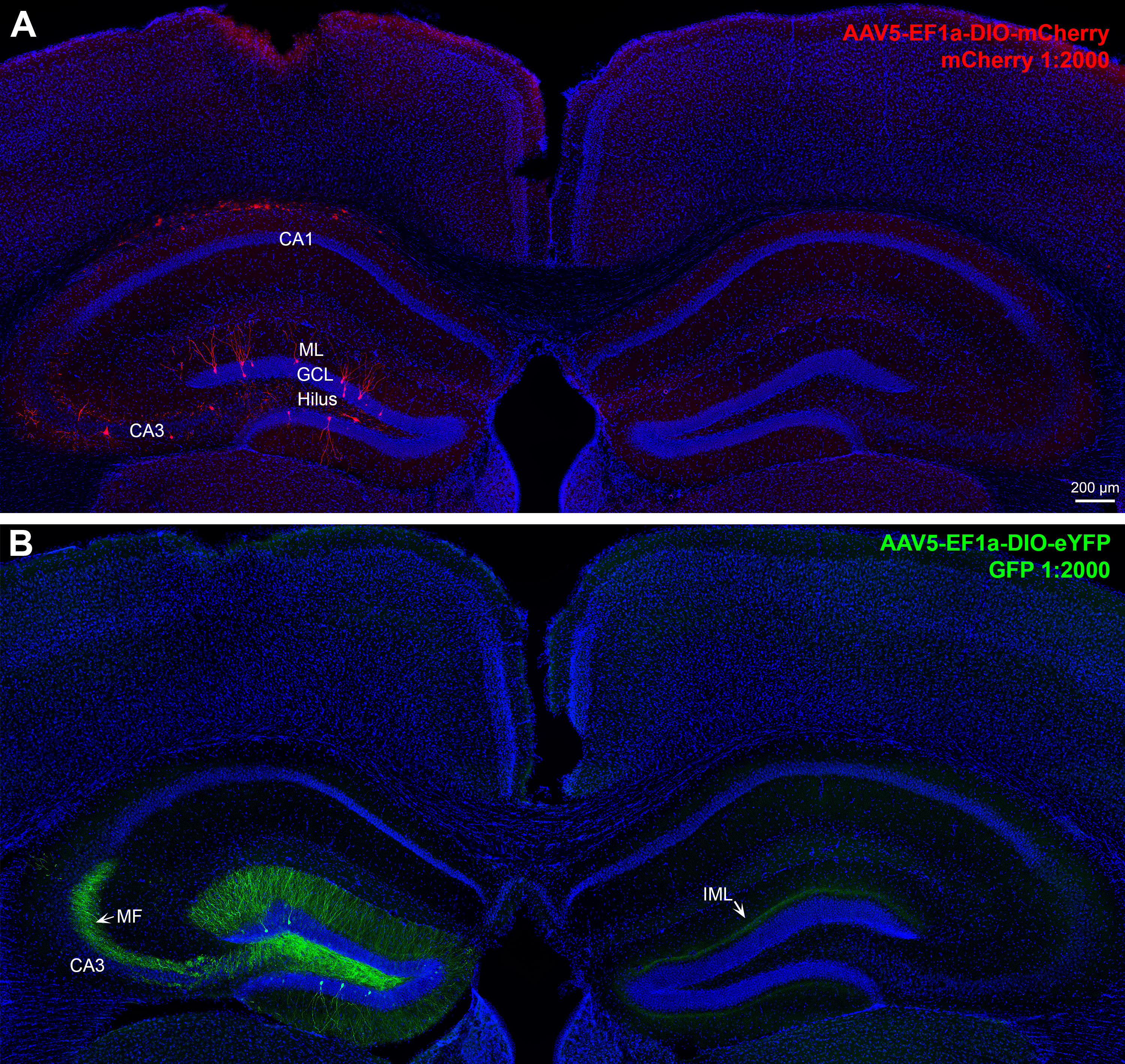

Supplement: Figure 4-1 — Fluorescence signal amplification of DIO-mCherry and DIO-eYFP is highly specific to the injection site in WT C57BL/6J mice. A, Tile scan of a C57BL/6J mouse injected with AAV5-EF1a-DIO-mCherry. Viral expression was amplified with mCherry antibody. The indent on the top of the left cortex represents a drilling artifact near the injection site. The mCherry expression is primarily restricted to the injected (left) hippocampus, with mCherry+ cells observed in the GCL of the DG. There is also sparse labeling of mCherry+ cells in the CA3. B, Tile-scan of a C57BL/6J mouse injected with AAV5-EF1a-DIO-eYFP. Viral expression was amplified with GFP and observed primarily within the injected (left) DG. Furthermore, GFP+ mossy fiber (MF) axons from dentate GCs were observed projecting to area CA3. Interestingly, commissural GFP+ axons, presumably from mossy cells, were observed within the IML of the contralateral hemisphere. Notably, there were no mCherry+ or GFP+ cells in the noninjected hemisphere. This result indicates that amplified fluorescence signal is highly specific to the target region and the projections of labeled cells. Scale bar, 200 μm. Download Figure 4-1, TIF file. [file enu-eN-NWR-0363-21-s03.tif]

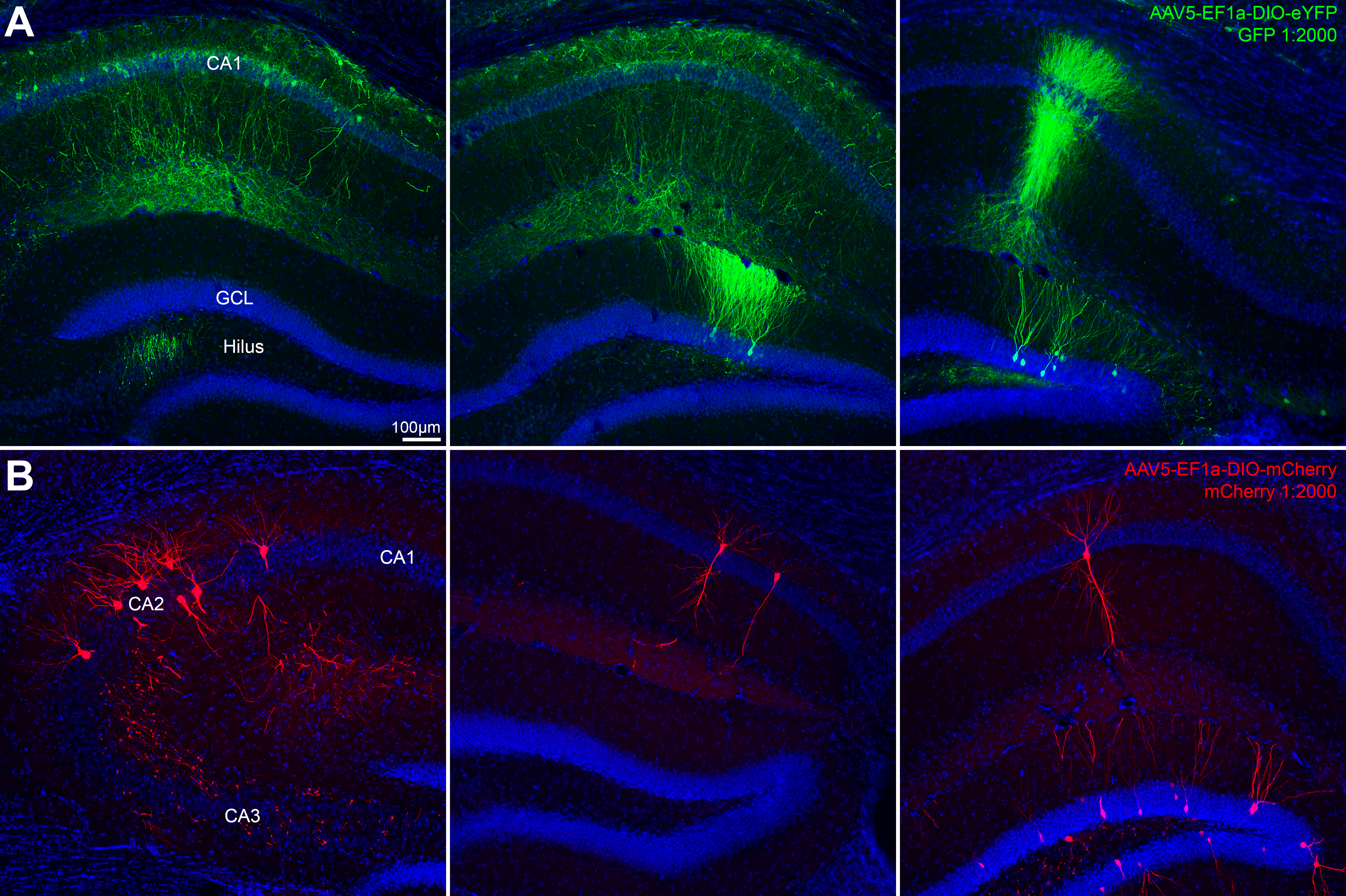

Supplement: Figure 4-2 — Fluorescence signal amplification in other subfields of the hippocampus. A, B, Viral injections aimed at the DG occasionally resulted in mistargeting, which led to amplified fluorescence signal in other subfields of the hippocampus, such as CA1 or CA2. This finding suggests that amplified viral expression was not unique to the DG, but rather was specific to the injection site. Scale bar, 100 μm. Download Figure 4-2, TIF file. [file enu-eN-NWR-0363-21-s04.tif]

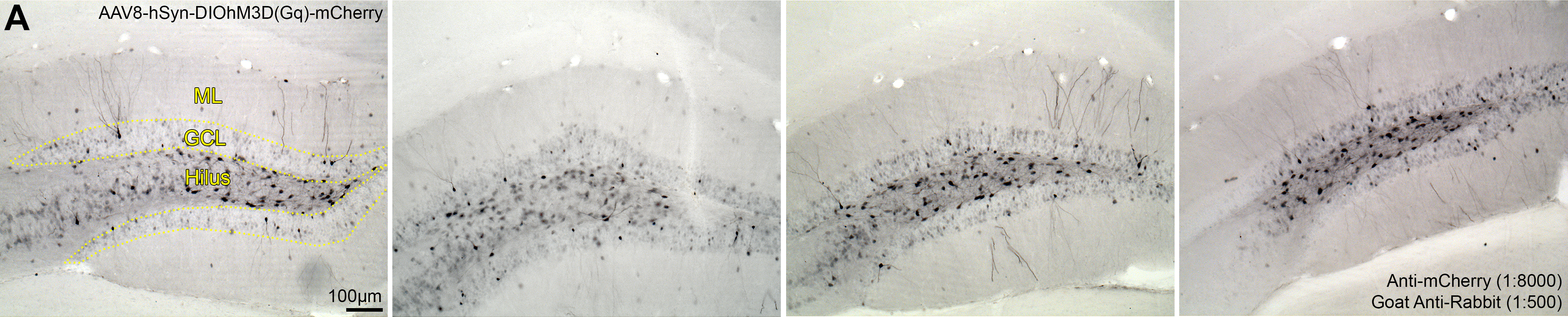

Supplement: Figure 6-1 — mCherry immunoreactivity in WT C57BL/6J mice injected with AAV8-hSyn-DIO-hM3Dq-mCherry. A, Representative photomicrographs of mCherry immunoreactivity in C57BL/6J mice injected with AAV8-hSyn-DIO-hM3Dq-mCherry. The pattern of mCherry immunoreactivity was comparable to the amplified immunofluorescence of DIO-hM3Dq-mCherry (Fig. 6). Scale bar, 100 μm. Download Figure 6-1, TIF file. [file enu-eN-NWR-0363-21-s05.tif]

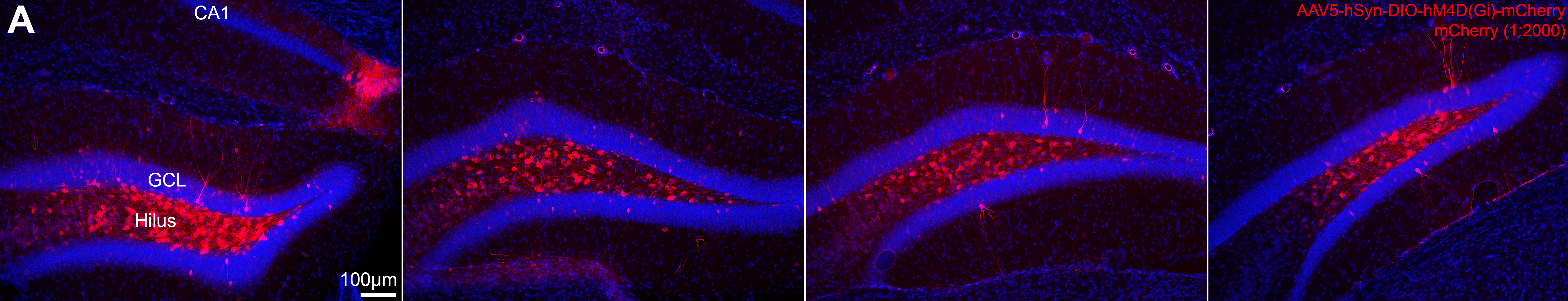

Supplement: Figure 6-2 — Fluorescence signal amplification of AAV5-hSyn-DIO-hM4Di-mCherry in WT C57BL/6J mice. A, C57BL/6J mice were injected in the DG with AAV5-hSyn-DIO-hM4D(Gi)-mCherry, and sections were amplified with mCherry. Interestingly, mCherry+ cells were primarily located in the hilus, but a small number of GCs were also labeled. The pattern of amplified AAV5-hSyn-DIO-hM4Di-mCherry expression is consistent with the AAV8-hSyn-DIO-hM3Dq-mCherry construct shown in Figure 6. Scale bar, 100 μm. Download Figure 6-2, TIF file. [file enu-eN-NWR-0363-21-s06.tif]
